# Interpersonal neural synchrony and mental disorders: unlocking potential pathways for clinical interventions

**DOI:** 10.3389/fnins.2024.1286130

**Published:** 2024-03-11

**Authors:** Kerstin Konrad, Christian Gerloff, Simon H. Kohl, David M. A. Mehler, Lena Mehlem, Emily L. Volbert, Maike Komorek, Alina T. Henn, Maren Boecker, Eileen Weiss, Vanessa Reindl

**Affiliations:** ^1^Child Neuropsychology Section, Department of Child and Adolescent Psychiatry, Psychosomatics and Psychotherapy, University Hospital RWTH, Aachen, Germany; ^2^JARA Brain Institute II, Molecular Neuroscience and Neuroimaging (INM-11), Jülich Research Centre, Jülich, Germany; ^3^Department of Applied Mathematics and Theoretical Physics, Cambridge Centre for Data-Driven Discovery, University of Cambridge, Cambridge, United Kingdom; ^4^Department of Psychiatry, Psychotherapy and Psychosomatics, Medical School, RWTH Aachen University, Aachen, Germany; ^5^Institute for Translational Psychiatry, University of Münster, Münster, Germany; ^6^School of Psychology, Cardiff University Brain Research Imaging Center (CUBRIC), Cardiff University, Cardiff, United Kingdom; ^7^Institute of Medical Psychology and Medical Sociology, University Hospital RWTH, Aachen, Germany; ^8^Department of Psychology, School of Social Sciences, Nanyang Technological University, Singapore, Singapore

**Keywords:** interpersonal neural synchrony (INS), hyperscanning neurofeedback, mental disorders, social dysfunction, brain stimulation

## Abstract

**Introduction:**

Interpersonal synchronization involves the alignment of behavioral, affective, physiological, and brain states during social interactions. It facilitates empathy, emotion regulation, and prosocial commitment. Mental disorders characterized by social interaction dysfunction, such as Autism Spectrum Disorder (ASD), Reactive Attachment Disorder (RAD), and Social Anxiety Disorder (SAD), often exhibit atypical synchronization with others across multiple levels. With the introduction of the “second-person” neuroscience perspective, our understanding of interpersonal neural synchronization (INS) has improved, however, so far, it has hardly impacted the development of novel therapeutic interventions.

**Methods:**

To evaluate the potential of INS-based treatments for mental disorders, we performed two systematic literature searches identifying studies that directly target INS through neurofeedback (12 publications; 9 independent studies) or brain stimulation techniques (7 studies), following PRISMA guidelines. In addition, we narratively review indirect INS manipulations through behavioral, biofeedback, or hormonal interventions. We discuss the potential of such treatments for ASD, RAD, and SAD and using a systematic database search assess the acceptability of neurofeedback (4 studies) and neurostimulation (4 studies) in patients with social dysfunction.

**Results:**

Although behavioral approaches, such as engaging in eye contact or cooperative actions, have been shown to be associated with increased INS, little is known about potential long-term consequences of such interventions. Few proof-of-concept studies have utilized brain stimulation techniques, like transcranial direct current stimulation or INS-based neurofeedback, showing feasibility and preliminary evidence that such interventions can boost behavioral synchrony and social connectedness. Yet, optimal brain stimulation protocols and neurofeedback parameters are still undefined. For ASD, RAD, or SAD, so far no randomized controlled trial has proven the efficacy of direct INS-based intervention techniques, although in general brain stimulation and neurofeedback methods seem to be well accepted in these patient groups.

**Discussion:**

Significant work remains to translate INS-based manipulations into effective treatments for social interaction disorders. Future research should focus on mechanistic insights into INS, technological advancements, and rigorous design standards. Furthermore, it will be key to compare interventions directly targeting INS to those targeting other modalities of synchrony as well as to define optimal target dyads and target synchrony states in clinical interventions.

## 1 Introduction

The evolution of humans as social creatures has prepared our brains to be ideally primed for interpersonal interactions (Hari and Kujala, 2009). When we naturally mirror each other’s smiles or laughter during social engagement or unintentionally align our body language with those we are speaking to, these are moments of behavioral synchrony. Moreover, interpersonal synchrony is evident in hormonal states and the autonomous nervous system (physiological synchrony), as well as in neural responses among two (or more) individuals interacting with each other (interpersonal neural synchrony, INS).

Measures of the autonomous nervous system (ANS) become synchronized in interacting dyads, reflecting the activity of the sympathetic and parasympathetic nervous system. This includes, e.g., heartbeat, electrodermal activity, or breathing rhythm (Davis et al., 2018; Bell, 2020). Furthermore, hormonal levels like oxytocin or cortisol get attuned in interacting dyads. This phenomenon can be seen from birth on and in the case, e.g., of parents and their infants critically depends on the quality of their behavioral synchrony (Feldman et al., 2011; Feldman, 2012b).

Interpersonal neural synchrony (INS) is the temporal relationship between two person’s brain signals while interacting and seems to reflect a fundamental mechanism of bi-directional attunement (Azhari et al., 2019). In particular, neural activity is coordinated in frontotemporal cortices when humans are interacting (see Lotter et al., 2023 for a review). INS can be captured by hemodynamic or electrophysiological measures. Electroencephalography (EEG) / magnetoencephalography (MEG), near-infrared spectroscopy (fNIRS), and functional magnetic resonance imaging (fMRI) are the most widely used imaging techniques to capture neural synchrony patterns of two or more subjects concurrently, which is called hyperscanning (Czeszumski et al., 2022).

INS is associated with both external and internal factors: External influences might include non-social triggers such as common sensory input, as well as social triggers like shared eye contact, language, or movement. On the other hand, internal synchronizers might encompass personality traits, the mental states of those interacting, the social closeness between them, and motivational states (Dikker et al., 2021).

Synchrony across all known modalities, such as in behavior, hormonal states, autonomous nervous and central nervous activity can be seen as a crucial underlying factor of social participation and social cohesion (Feldman, 2017). Being able to adaptively get “in-sync” and “out-of-sync” with others may represent important requirements for successful interaction in terms of communication, social bonding, and affiliation (Hove and Risen, 2009; Rennung and Göritz, 2016; Vicaria and Dickens, 2016; Mogan et al., 2017; Hu et al., 2022). Some of these requirements are the individuals’ adaptive capacities to access another’s internal arousal state (Mizugaki et al., 2015), share and regulate emotions (Davis et al., 2018; Birk et al., 2022), to learn from each other (Pan et al., 2021), and to adapt to collective behaviors and group norms (Wiltermuth and Heath, 2009; Reinero et al., 2021).

A lack of social interaction abilities and maladaptive relationships during the early formative years as well as throughout the life span are among the most significant factors leading to mental disorders (Schilbach, 2016; Schilbach and Lahnakoski, 2023). Vice versa, mental disorders can affect our abilities to successfully interact with others and to enjoy social interactions. Psychotherapy, an effective treatment for many mental disorders, utilizes the structured therapeutic relationship to favor the patients’ well-being. Thus, it has been suggested that mental disorders in general can be construed as disorders of social interaction (Schilbach, 2016). However, there are some mental disorders, such as autism spectrum disorder (ASD), (Reactive) attachment disorder (RAD), or social anxiety disorder (SAD), that are particularly characterized by disruption of social interaction and communication as an essential part of their underlying patho-mechanisms. In line with this, there is first evidence derived from recent hyperscanning studies that decreased INS is associated with (i) increased level of social difficulties in everyday life in subjects with ASD (Quiñones-Camacho et al., 2021), (ii) predictive of poor attachment quality in high-risk mother–child dyads (Miller et al., 2019) and (iii) linked to symptom severity in SAD when assessed in emotionally negative situations (Deng et al., 2022).

However, despite the compelling evidence for the broad relevance of social interaction in mental health and the emerging evidence for a specific role of synchrony as an important underlying mechanism, so far, the majority of neuroscientific studies focused on the mechanisms, antecedents, and consequences of INS under typical and atypical conditions largely ignoring INS as a therapeutic target for mental health interventions. However, the recent technical advances in the field of hyperscanning, real-time neurofeedback, and neurostimulation techniques open up new avenues to translate this second-person neuroscience approach into interactive-based neuroscientific interventions. Tackling directly the neural mechanism involved in social function and dysfunction might thus help to develop novel effective prevention strategies and interventions for a variety of mental disorders.

**Figure 1:**
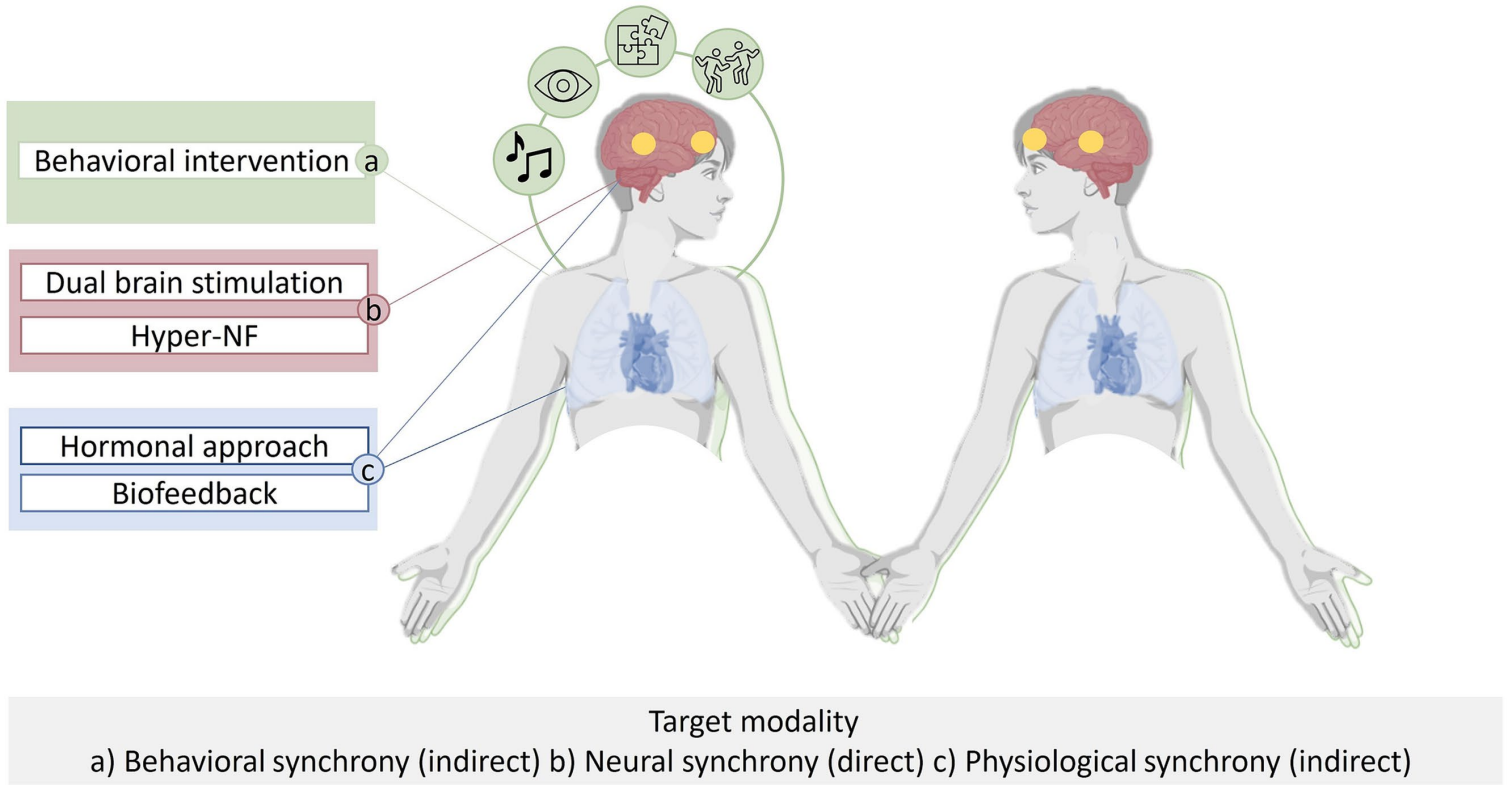
Interpersonal Synchrony occurs at multiple levels (i.e., behavior, autonomous nervous system, hormones and brain) in interacting subjects. Interpersonal neural synchrony (INS) can be targeted through *direct* manipulation (depicted in RED) via dual brain stimulation or hyperscanning neurofeedback (hyper-NF). INS can be targeted *indirectly* by behavioral interventions (depicted in GREEN), or by peripher-physiological biofeedback or pharmacological approaches (depicted in BLUE).

In principle, a range of methods can be employed to influence INS. Neural synchrony can be targeted through direct manipulation or indirectly by targeting alternative modalities of synchrony to enhance INS. Directly INS can be targeted using brain stimulation and hyperscanning neurofeedback (hyper-NF), indirectly it can be targeted using pharmacological approaches, ANS-biofeedback and behavioral interventions aiming to enhance INS (Figure 1).

In this work, we will outline these translational approaches and provide an overview how they impact on INS. We will systematically review those approaches that directly target INS by (i) hyper-NF and (ii) neurostimulation approaches, and (iii) how well such approaches are generally accepted by patients. Furthermore, we will exemplify how manipulation of INS can contribute to treatment of three selected mental disorders, all characterized by social interactions deficits, ASD, RAD and SAD. While here we highlight these three mental disorders, we note that INS-based approaches may also be relevant to other psychiatric or neurological diseases that are characterized by impaired social interactions. Finally, we will provide a critical outlook on open research questions, technical challenges, and clinical caveats to be considered in future studies.

## 2 Methods

In this review, we offer a concise overview of indirect strategies for manipulating INS, while also presenting a comprehensive state-of-the-art illustration of all translational techniques that directly target INS. The latter insights are derived from systematic database searches, providing a thorough analysis of the current landscape. To foster discussions about progress towards clinical utility, open technical challenges for these novel approaches will be outlined. Finally, we will narratively review promising clinical applications for improving interaction-based mental health outcomes in the future along with a systematic review on their general acceptability by patients. Of note, in the clinical translation part we will exclusively focus on future avenues for INS-based approaches for treatment of social interaction disorder while leaving out the scope in terms of INS-based diagnostics or patient stratification.

To provide a comprehensive overview of techniques directly targeting INS and assess their feasibility and acceptability, we conducted three structured literature searches (i: neurostimulation, ii: hyper-NF, iii: feasibility / acceptability). Literature searches were conducted using the open-source search engine SetYouFree (v. 0.1.2, https://github.com/ChristianGerloff/set-you-free, Gerloff et al., 2022b) following the best practices of the PRISMA guidelines for systematic reviews. As the reviewed techniques are in their early stages, the search was conducted across scientific publication databases as well as preprint servers. All searches were performed starting from 1st January 2000 up to the 31th December 2023 (date of conduction of ii), which includes the first hyperscanning studies (Montague et al., 2002). Publications were screened in SetYouFree using deterministic exclusion criteria, which were *a-priori*-defined (see Supplementary Tables S1–S3). For one of the searches (ii), the screening was conducted by two independent reviewers to assess their interrater reliability (Cohen’s kappa = 0.95). To ensure reproducibility, technical details, parameters, search flows and human readable SetYouFree files are provided in the Supplementary materials (Text “Structured Literature Search”, Supplementary Tables S1–S3, Supplementary Figures S1–S3).

To guide the reader, the kind of search (systematic or unsystematic) is explicitly described at the beginning of each paragraph in the Result section. In Section 3.2.1 we provide an overview of brain stimulation results based on a systemic literature search using search strings that combined “neurostimulation”, “TMS”, “tDCS” or “tACS” with hyperscanning-related terms. From the initial pool of *N* = 695 studies, we identified seven studies that applied tACS (*N* = 5), tDCS (*N* = 1) or both (*N* = 1) in one or both members of a dyad with the goal to manipulate INS and examining changes in behavioral and/or neural synchrony.

In Section 3.2.2 we investigated the general feasibility and training success of hyper-NF based on twelve eligible publications (EEG: *N* = 10, fNIRS: *N* = 1; fMRI: *N* = 1) out of 129 initial search results, using a search string that combined “neurofeedback” with hyperscanning-related terms (see i).

Finally, in Section 3.4 we analyzed the general acceptability and feasibility of direct intervention techniques in patients with social interaction dysfunction across 8 out of 321 studies based on neurofeedback (*N* = 4) and neurostimulation (*N* = 4). For this search, we combined the search terms from (i) and (ii) with relevant disorder related terms (“social”, “autism”, “attachment”) and outcome specific terms (“acceptability”, “feasibility”, “acceptance”, “compliance”, “adherence”). The specific search terms are provided in Supplementary Tables S1–S3.

## 3 Results

### 3.1 Techniques targeting INS indirectly through other modalities of synchrony

One of the most widely used indirect approaches to target INS is through behavioral techniques that aim to modify behavior and foster shared experiences and emotional bonding. Hyperscanning studies using EEG/MEG, fNIRS, and fMRI have shown increased INS in a variety of social-interactive tasks, e.g., emotional sharing, joint action, coordinated and synchronized movement, communication, making music, eye contact and joint attention, cooperation and competition, decision making and learning tasks (e.g., see Nam et al., 2020 for a review). Based on these studies, we will non-systematically summarize several behavioral techniques that may be particularly well-suited for increasing INS and promoting positive outcomes in Section 3.1.1. Given the close link between the brain and other bodily systems, INS may additionally be enhanced by targeting synchrony in the ANS and by hormonal manipulations. These techniques will be non-systematically summarized in Section 3.1.2.

#### 3.1.1 Behavioral techniques

Some promising candidates for enhancing INS include (i) coordinating or synchronizing body movements, (ii) making music together, (iii) playing cooperative or interactive games, and (iv) engaging in shared eye contact or joint attention. Of note is that while these possibilities are not the exclusive ones, they are highlighted here because there is fairly robust evidence for all four of them.

##### 3.1.1.1 Coordinated and synchronized movements

Evidence suggests that coordinated or synchronized body movements are associated with increased INS. Examples of tasks that induce INS include synchronized button press after counting a time in mind (Hu et al., 2017), synchronized arm movement (Nozawa et al., 2019) and imitation tasks (Dumas et al., 2010; Holper et al., 2012; Miyata et al., 2021), as well as many cooperative tasks that involve action coordination or synchronization (e.g., Cui et al., 2012; Li et al., 2020; see below for more information). Further, experimental manipulations of movement synchrony have been shown to induce higher affiliation (Hove and Risen, 2009), altruistic and prosocial behavior (Valdesolo and DeSteno, 2011; Cirelli et al., 2014), cooperation (Wiltermuth and Heath, 2009) and joint action performance (Valdesolo et al., 2010). Some of these effects may be facilitated through increased INS (Hu et al., 2017). While most hyperscanning studies have measured the effects of behavioral synchrony on INS concurrently, Nozawa et al. (2019) have probed the prolonged effects of an experimental movement synchrony manipulation on subsequent social interaction. In their study, pairs of participants had to move their arm to a beat of a sound, presented either at the same or at a different tempo. After the rhythmic movement block, participants engaged in an educational communication where they taught and learned unknown words to/from each other during which their neural activities in medial and left lateral prefrontal cortices were measured using fNIRS hyperscanning. Results showed that prior movement synchrony enhanced both teacher-learner rapport and INS in the left lateral prefrontal cortex in the subsequent teaching-learning task, and these changes were interrelated. Thus, although these findings need to be replicated, this suggests that movement synchrony induction can have at least short-term carry-over effects on social interaction.

##### 3.1.1.2 Cooperative behavior and games

As shown by a recent systematic review and quantitative meta-analysis, cooperative behavior has been associated with statistically significant INS, with a large overall effect sizes in frontal and temporoparietal areas, across diverse cooperative tasks, including Jenga game, Tangram puzzle, creativity tasks, joint finger tapping, drawing and singing, realistic problem solving and a math task (*N* = 13 fNIRS-based hyperscanning studies with 890 human subjects; Czeszumski et al., 2022). Many of the cooperative tasks have been applied or are potentially suitable for children and adolescents (e.g., Reindl et al., 2018, 2022; Kruppa et al., 2021). Importantly, compared to simple movement coordination/synchronization tasks, gamifying behavioral synchrony induction tasks (e.g., as in Cui et al., 2012 or Reindl et al., 2018) may make the tasks more engaging, particularly for developmental populations, and thus potentially more suitable for behavioral interventions.

##### 3.1.1.3 Music

Singing or humming together, drumming, playing musical instruments like guitar or piano, musical improvisation and listening to music have been associated with INS in multiple studies (e.g., Lindenberger et al., 2009; Sänger et al., 2012; Müller et al., 2013, 2018; Osaka et al., 2015; Zamm et al., 2018; Müller and Lindenberger, 2019, 2022, 2023; Hou et al., 2020; Liu et al., 2021; Chabin et al., 2022; Gugnowska et al., 2022). The close link between music and INS is not surprising given that making music involves sensorimotor coupling in musicians, being entrained to the same pace and rhythm as well as coordinating and synchronizing actions. Further, music is a pleasurable experience that is linked to emotional sharing in musicians, audience as well as between musicians and audience. In addition to its emotional effects, it promotes social functions, such as communication, cooperation, and social attachment, and thereby might possess inherent therapeutic potential (Koelsch, 2014).

##### 3.1.1.4 Eye contact

Irrespective of the specific task, ostensive signals have been proposed to entrain oscillatory brain signals during the social interaction (e.g., via mutual phase resetting in sender and receiver; Wass et al., 2020). Ostensive signals are cues that a communicator uses to convey their communicative intention to an addressee (Wass et al., 2020); one of the most notable is eye contact. Studies in adults and adult-child dyads show that eye contact elicits increased INS (e.g., Dikker et al., 2017; Hirsch et al., 2017; Koike et al., 2019; Dravida et al., 2020; Noah et al., 2020; Guglielmini et al., 2022; Luft et al., 2022; but see Haresign et al., 2023 for contradictory findings). This has been demonstrated both in naturalistic tasks, e.g., during communication (Piazza et al., 2020), as well as in well-controlled experimental set-ups, e.g., eye-to-eye contact compared to mutual gaze at the eyes of a picture face (Hirsch et al., 2017). Further, Dikker et al. (2017) demonstrated that engaging in a short eye-to-eye contact activity could serve as an intervention to enhance subsequent INS. In their study, the EEGs were recorded of 12 students simultaneously over the course of a semester during regular classroom activities. Prior to the class, students engaged in a 2-min eye contact with an assigned peer. Students showed highest INS with their face-to-face partner during class, which was correlated with student’s mutual closeness rating. Such a correlation was only observed for face-to-face partners; thus, it seemed to “activate” interpersonal relationship features.

Taken together, behavioral studies indicate that social interactions that involve coordinating or synchronizing actions, cooperation, music and eye contact are associated with increased INS, and thus are potentially suitable as behavioral interventions. However, the majority of studies have evaluated INS during the behavioral intervention or experimental condition. Only a very limited number of studies have explored the effects of behavioral interventions on INS measured shortly thereafter, indicating that these tasks may have carry-over effects (Dikker et al., 2017; Nozawa et al., 2019; Khalil et al., 2022). While long-term effects on INS manipulations are rather unexplored, some of these techniques have been implemented in therapeutic approaches (e.g., in a dance/movement interventions based on interpersonal movement imitation and synchronization; Koehne et al., 2016) (for further information see Section 3.3). Nevertheless, before moving behavioral synchrony manipulations towards clinical application in patients with social dysfunction more research is needed to demonstrate whether they do show longer-lasting effects on social interactive behaviors, INS or both.

#### 3.1.2 ANS-based biofeedback and hormonal manipulations

Another indirect technique to manipulate synchrony in interacting individuals is to provide feedback systems to measures of the ANS. This form of biofeedback training can for instance be provided based on heart rate variability. As such, it can not only be employed in single-person designs to entrain self-regulation of the underlying biological signal (Mather and Thayer, 2018), but is also considered a suitable approach for dyadic contexts to increase interpersonal synchrony. For example, it may help to increase interpersonal synchrony in a therapeutic setting (Fiskum, 2019) or promote empathy and social entrainment between two people (Tennant et al., 2019). Besides heart rate variability, skin conductance has been used to provide (simulated) dyadic feedback (Feijt et al., 2020) and other studies have provided ANS-based biofeedback based on breathing rhythm (Järvelä et al., 2019, 2021; Stepanova et al., 2020; Salminen et al., 2022). Popular feedback modalities in this context are pure visual (Feijt et al., 2020), or auditory feedback (Tennant et al., 2019) as well as a multisensorial approach via virtual realities (Järvelä et al., 2019, 2021; Stepanova et al., 2020; Salminen et al., 2022).

Along with biofeedback, hormonal approaches can be employed to intentionally enhance INS. Notably, maternal chemo-signals have been found to heighten INS between parents and infants and can also foster increased INS between an infant and an unfamiliar person (Endevelt-Shapira et al., 2021). Moreover, studies have demonstrated that the administration of intranasal oxytocin can effectively boost INS (Mu et al., 2016) and promote behavioral synchrony in adult participants (Spengler et al., 2017). Again, nothing is known about any longer-lasting effects of such manipulations.

### 3.2 Techniques directly targeting INS

Promising manipulation approaches that target synchrony directly on the neural level include brain stimulation through methods like transcranial direct current stimulation (tDCS) or transcranial alternating current stimulation (tACS), as well as hyper-NF. These techniques aim to directly influence INS in the brain.

Both tDCS and tACS pass a low electrical current through the brain to modulate neural activity (Kropotov, 2016) and can be used to influence the activity of specific brain regions, such as regions that are involved in social cognition and interpersonal interaction. Specifically, tDCS delivers a weak, direct current to the brain through scalp electrodes and can thereby manipulate the membrane potential of neurons and modulate spontaneous firing rates, producing facilitatory or inhibitory effects upon a variety of behaviors (Paulus, 2011; Thair et al., 2017). Instead of using a direct current, tACS delivers sinusoidally varying transcranial stimulation that may interact with ongoing rhythms in the cortex (Paulus, 2011). tDCS may create a stable environment that predisposes neural circuits towards synchronization, potentially affecting INS through sustained shifts in excitability and plasticity. tACS, on the other hand, could immediately and dynamically entrain neural oscillations and directly modulate the temporal dynamics of brain activity, providing a more immediate but possibly less enduring impact on INS. Thus, while both, tDCS and tACS can modulate INS, they may do so through different mechanisms and with different temporal dynamics. tDCS might promote a general state of heightened plasticity and readiness for synchronization, while tACS could directly entrain and synchronize oscillatory activity in real-time, which is typically considered the foundation of INS (Lu et al., 2023). In comparison, tDCS applies a constant stimulation and can thus modulate brain activities in regions associated with the mental processes that are being probed. tDCS can also reduce INS, e.g., by applying stimulation to one participant but not the other (Long et al., 2023). The influence of those two brain stimulation techniques has been shown to impact on INS in dyads with respect to synchrony in communication (Long et al., 2023), movement (Novembre et al., 2017; Szymanski et al., 2017; Pan et al., 2021) as well as enhanced learning performances (Pan et al., 2021).

While brain stimulation influences the brain signal most directly, neurofeedback allows participants to intentionally manipulate their neural activity through real-time information about their brain activity (Marzbani et al., 2016). Recently, initial studies have focused on hyper-NF also called dual neurofeedback or so-called cross-brain neurofeedback (Duan et al., 2013; Zhang and Zhao, 2018). In this context, mainly EEG (Järvelä et al., 2019, 2021; Dikker et al., 2021; Müller et al., 2021; Putri et al., 2022; Salminen et al., 2022; Ceccato et al., 2023; Vrins et al., 2023) but also fNIRS (Duan et al., 2013) and fMRI (Kerr et al., 2022) have been used.

So far, little is known about how direct approaches can be used to manipulate INS intentionally, even though brain stimulation and hyper-NF are promising techniques and represent two of the most suitable, practicable, and relatively cost-effective methods for clinical translation. Therefore, we will provide a systematic overview of the current literature on brain stimulation approaches in section 3.2.1 and hyper-NF in Section 3.2.2. Additionally, we will discuss considerations of optimizing technical and design issues for hyper-NF (Boxes 1, 2).

BOX 1 **Methodological considerations for hyper-NF.**

From the reviewed hyper-NF studies, we will explore key methodological and design considerations, aiming to provide a comprehensive overview of the selected parameters and methodological considerations.

Typically, in neurofeedback, predefined brain regions are selected that have been shown to be associated with the psychological or biological construct of interest to calculate the feedback. The selection of these target regions varies considerably across studies. Some studies focus on distinct parietal regions targeted by a single EEG channel (Susnoschi Luca et al., 2021; Putri et al., 2022) while others consider multiple regions, such as the left and bilateral sensorimotor cortex in motor-related experiments (Duan et al., 2013; Zhang and Zhao, 2018). Dikker et al. (2021) and Vrins et al. (2023) performed whole-brain-based neurofeedback. Notably, all studies targeted only homologous brain regions between participants in a dyad or averaged the signals across regions.

The neural recordings from these targeted regions are subsequently online preprocessed. Online preprocessing can be performed either directly on an infinite stream of data or by slicing a finite number of subsequent samples into batches, termed batch processing (Marz and Warren, 2015). The reported batch sizes varied considerably across studies, ranging from 0.5 s up to 9 s for EEG experiments. Increasing the overlap of batches allows for a higher refresh frequency (the frequency at which the preprocessed signal is updated) of the preprocessed signal, approaching the sampling frequency. The refresh frequency in the reviewed studies ranged from 0.5 Hz in fMRI up to 60 Hz in EEG, enabling continuously perceived visual feedback. Signal processing operations on these batches involved signal averaging across target regions and time, transformations (e.g., Hilbert Transform), and applying filters, e.g., to select the desired frequency band in EEG studies. The choice of frequency band differed across EEG studies. For instance, Müller et al. (2021) targeted both the theta band and the delta band in separate training runs. In fNIRS, an important methodological consideration pertains to the signal type since fNIRS measures changes in oxyhemoglobin (HbO) and deoxyhemoglobin concentrations in the brain. Duan et al. (2013) targeted HbO to derive feedback features, a common approach in fNIRS neurofeedback studies (see Kohl et al., 2020). Notably, none of the studies applied bad channel detection or artifact correction beyond filters (see Nolan et al., 2010; Mullen et al., 2015; Aranyi et al., 2016; Kohl et al., 2023).

**Figure 2:**
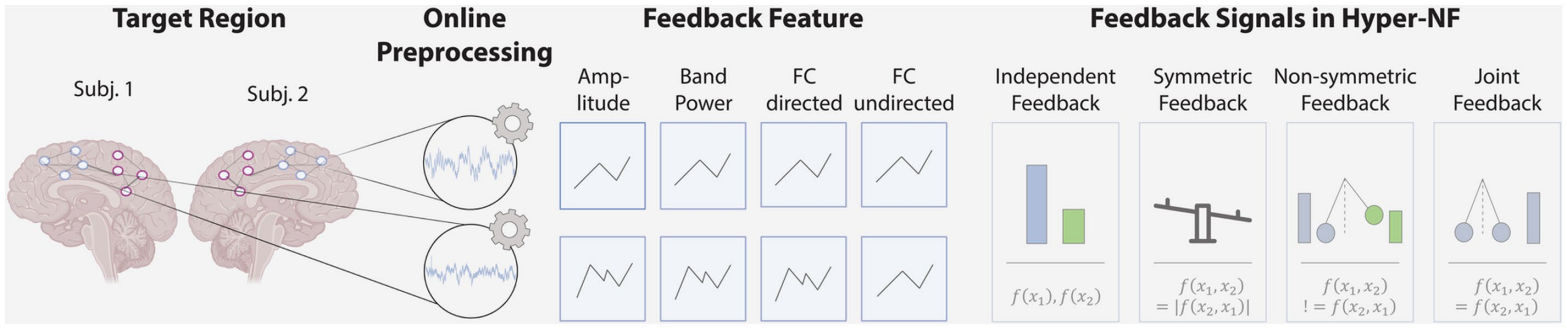
Study design considerations. Hyper-NF begins with the observation of neural activity of multiple persons using EEG/MEG, fNIRS or fMRI in target brain regions or network of regions. In an online analysis, signals are preprocessed and a feedback signal is calculated based on features such as the signal’s amplitude, band power or directed / undirected functional connectivity. This is then fed back to the participants through visual, auditory, or other modalities using independent, symmetric, non-symmetric or joint feedback.

Based on the preprocessed signals, a single or a set of features are derived, forming the basis of the subsequently presented feedback. The features in the studies reviewed here can be differentiated into three categories: amplitude and power-based estimators, or functional connectivity estimators. Both amplitude and power-based feedback provide independent metrics for each subject ( $f\left(x_{1}\right),f\left(x_{2}\right)$ ). These can be feedbacked independently, e.g., as two bars, or a difference between subjects’ features can be calculated and presented to the participants, e.g., as a see-saw, resulting in symmetric feedback ( $f\left(x_{1},\;x_{2}\right)$ = |f(x2,x1)| , see Figure 2) In contrast to amplitude and power-based feedback features, connectivity estimators quantify synchrony directly $(f\left(x_{1},\;x_{2}\right)$ ). Here, generally, non-directed and directed estimators can be differentiated (Bastos and Schoffelen, 2016). While non-directed metrics aim to capture some form of interdependence between signals, without considering any direction of influence, directed metrics seek to establish a statistical causation from the data that is based on the principle that cause precedes effect (e.g., from parent to child or vice versa). Among the reviewed studies, only undirected functional connectivity estimators were used. These undirected estimators, such as the Pearson correlation, provide a single value that is equal for both participants ( $f\left(x_{1},\;x_{2}\right)$ = $f\left(x_{2},x_{1}\right)$ ). This value indicating high or low INS is feedbacked to the participants, e.g., as a pendulum (joint feedback; Figure 2). In contrast, directed FC, such as Granger causality, can provide individual feedback for each person which is dependent but non-symmetric ( $f\left(x_{1},\;x_{2}\right)$ != $f\left(x_{2},\;x_{1}\right)$ ), thus allowing to remove individual training differences within a dyad. The feedback signal should fit the task at hand. For example, joint feedback may be less applicable for a competitive task where the winner should be displayed, while in a cooperative task both joint and individual feedback can provide valuable information.

To quantify success based on the feedback signal, either the continuous feedback signal itself can be used, for example, in an offline analysis, or a discrete performance measure can be calculated from the feedback signal. For instance, three studies thresholded feedback signals to estimate discrete states of synchronization or desynchronization, and then counted the occurrence of the desired state if the state lasted over a period, such as 1 s (Müller et al., 2021; Susnoschi Luca et al., 2021; Putri et al., 2022).

BOX 2 **Design considerations for hyper-NF.**

Conceptually, cooperative, and competitive tasks can be differentiated. Cooperative tasks typically reinforce a synchronization of neural activity between dyads. In contrast, competitive tasks reinforce a desynchronization, e.g., by targeting the upregulation of one’s neural activities above those of the opponent, or by targeting neural activities into opposite directions (i.e., bidirectional training). However, whether indeed competition leads to lower, or possibly higher, INS than cooperation remains to be explored. Most studies employed a cooperative task, such as dyadic meditation (Järvelä et al., 2019, 2021; Salminen et al., 2022) or generating live music together (Ceccato et al., 2023; Vrins et al., 2023), while other studies employed competitive tasks, such as tug-of-war games (Duan et al., 2013; Zhang and Zhao, 2018). Depending on the task and neurofeedback target region, studies provide either specific instructions, e.g., motor imagery to regulate motor areas (Duan et al., 2013; Zhang and Zhao, 2018), or empathic, warm and compassionate feelings to regulate prefrontal EEG frequency bands (Järvelä et al., 2019, 2021; Salminen et al., 2022), or rather loose instructions as in Müller et al. (2021) who provide a few exemplary strategies but encourage a trial-and-error approach. A conceptional exception is the study by Kerr et al. (2022), in which mothers are instructed to regulate not their own brain activation but that of their daughters.

The presentation of feedback may differ with regard to (1) sensory modality (e.g., visual, auditory), (2) timing (immediate vs. delayed), (3) complexity (simple vs. complex), (4) reward (reward vs. no reward, type and time point of reward, see also Kohl et al. (2020). In previous hyper-NF studies, the feedback animations ranged from very simple visual designs such moving bar designs (Kerr et al., 2022) or intuitive approaching-ball-, pendulum- or seesaw-designs reflecting the IBS of both participants (Müller et al., 2021) to complex immersive audiovisual VR environments of a campfire scene using glowing connecting bridges between two avatars (Järvelä et al., 2019, 2021; Salminen et al., 2022). In the artistic approach of Dikker et al. (2021) participants sat face-to-face in a dome-like neurofeedback environment that immerses pairs of participants in a real-time audiovisual (AV) reflection of their EEG signals. In the study by Ceccato et al. (2023) and Vrins et al. (2023) participants received musical feedback. The pitch, intensity and pleasantness of the tone were determined by the different EEG features of the participants. Notably, nearly all studies provided immediate feedback on brain activity or synchrony. None of the studies provided delayed or post-block feedback, i.e., feedback after regulation. Two studies (Susnoschi Luca et al., 2021; Putri et al., 2022) presented additional rewards in the form of points that participants could collect throughout the training. Notably none of the studies used a form of social or monetary reward, which may boost regulation performance (Mathiak et al., 2015; Sepulveda et al., 2016; Kohl et al., 2023).

A hyper-NF protocol can be organized into trials/blocks, nested in runs, nested in sessions, although it should be noted that taxonomies differ between hyper-NF studies. A training run can be defined as a sequence of trials or blocks which is presented once or several times in one session, whereas different sessions are conducted on different days. Past protocols consisted of several training runs (1–8) on each day/session and up to six regulation trials per run. Besides the study by Susnoschi Luca et al. (2021) and Putri et al. (2022) (3 sessions) all studies employed singles-session training regimes. Whereas studies based on hemodynamic neuroimaging followed blocked designs with alternating regulation and resting/baseline blocks of 40 s each (Duan et al., 2013; Kerr et al., 2022), EEG studies involved longer regulation trials/blocks without breaks in between up to 5 min (Susnoschi Luca et al., 2021; Putri et al., 2022) or with breaks only after 5–10 trials (Müller et al., 2021), but resting baselines before the experiment, which was used for normalization purposes (Susnoschi Luca et al., 2021; Putri et al., 2022).

Neurofeedback experiments can employ several different control conditions (Sorger et al., 2019). Most studies were lacking a control condition, while some used sham or implicit feedback (i.e., participants were unaware that they received neurofeedback) (Dikker et al., 2021), no feedback, solo-neurofeedback or biofeedback (Järvelä et al., 2019, 2021; Salminen et al., 2022) and fake or inverted feedback (Müller et al., 2021). The implementation of random sham feedback can be challenging. For example, in Vrins et al. (2023), participants anticipated whether they were assigned to the control group and reported that the sham feedback was perceived as noisy music. Although no statistical comparison between cooperation and competition was conducted, the approach proposed by Susnoschi Luca et al. (2021) offers conceptually a bidirectional control mechanism that involves for one party a collaborative task to reinforce synchronization and for the other party a competitive task to reinforce desynchronization.

#### 3.2.1 Brain stimulation

Seven studies were identified that used brain stimulation protocols with the goal to modify INS and measure its effects on behavioral outcomes, with five studies using tACS (Novembre et al., 2017; Szymanski et al., 2017; Pan et al., 2021; Chen et al., 2022; Liu et al., 2023), one study using tDCS (Long et al., 2023) and one study using both (Lu et al., 2023) (see Table 1 for an overview). In a hyper-tACS protocol, the brains of a pair of participants are simultaneously stimulated and a behavioral outcome, such a movement synchrony, is measured during the stimulation. The stimulation can be applied with the same or different frequency and with the same or different phase. While same-phase-same-frequency stimulation is expected to enhance INS, different-phase-same-frequency or different-phase-different-frequency stimulation are expected to reduce INS, although it should be noted that INS was not actually measured in three hyper-tACS studies. In the first study, Novembre et al. (2017) showed that in-phase 20 Hz stimulation over the participants’ left motor cortices (same-phase-same-frequency) enhanced interpersonal movement synchrony in a finger tapping task, compared with anti-phase or sham stimulation, particularly for the initial taps following a preparatory period. This effect was specific for 20 Hz (beta oscillations) stimulation and was not found for 10 Hz (alpha oscillations) or 2 Hz (finger tapping frequency). In contrast to these findings, Szymanski et al. (2017) demonstrated that same-phase-same-frequency and different-phase-different-frequency stimulation over right frontal and parietal sites in the theta frequency range were associated with greater dyadic drumming asynchrony relative to a sham condition. This indicates that artificial modulation of inter-brain synchronization can actually impair rather than improve joint action coordination and highlight the importance of finding the optimal stimulation protocol to enhance synchrony. Investigating the potential of brain stimulation for social learning, Pan et al. (2021) found that tACS stimulation of learners and instructors over inferior frontal brain regions with in-phase 6 Hz alternating currents (same-phase-same-frequency) led to spontaneous and synchronized body movements and enhanced song learning performance (intonation accuracy) compared to a sham condition. Interestingly, the effects of in-phase stimulation on learning performance were partially mediated by interpersonal movement synchrony, suggesting that brain stimulation can facilitate learning through enhancing interpersonal synchrony. Similarly, Chen et al. (2022) found an increased gamma-band INS in left temporoparietal region for successful versus unsuccessful conceptual alignment. This was paralleled by an observed enhancement in conceptual alignment when gamma-band in-phase tACS was applied. Notably, these findings were derived from two distinct experiments. Liu et al. (2023) used a semiotics paradigm in which participants had to establish a novel interpersonal symbolic communication system using arbitrary symbols and figures prior to which the two brains were stimulated simultaneously using either 40 Hz in-phase, 40 Hz anti-phase or sham stimulation targeting the right superior temporal gyrus (rSTG). Throughout the stimulation period and communication task, brain activities were measured using fNIRS. Results showed that in-phase stimulation not only enhanced INS in the rSTG, but also improved communicative accuracy compared to the sham or anti-phase stimulation. Importantly, higher INS in the rSTG was observed both during the stimulation and task periods for in-phase compared to anti-phase and sham stimulation, showing that it was effective in increasing INS.

**Table 1:** Brain stimulation study results.

Author	*N* dyads	Type of stimulation	Stimulation conditions	Stimulation target	Task	Outcome	
						Neural	Behavioral
Chen et al. (2022)	27 (experiment 2)	Dual-tACS	40 Hz in-phase, sham (between-subjects)	Left STG	tACS during rest and semiotic game	NA	No significant differences in number of people in success / failure group; Accuracy higher for tACS vs. sham in success but not failure group
Liu et al. (2023)	70 (experiment 3)	Dual-tACS	40 Hz in-phase, 40 Hz anti-phase, sham (between-subjects)	Right STG	tACS prior to coordinating symbolic communication task	Higher INS in right STG during baseline and task for in-phase compared to anti-phase and sham stimulation	Communicative accuracy higher for in-phase compared to sham and anti-phase stimulation
Long et al. (2023)	30	Single tDCS	True, sham and control stimulation (within-subjects)	*True / sham*: Right ATL; *Control*: Occipital lobe	tDCS applied to *one* member of the dyad (women) prior to naturalistic communication task	Decreased INS for true compared to the sham and control stimulation	No significant differences in verbal or nonverbal behaviors; Reduced emotional empathy for true compared to sham and control stimulation
Lu et al. (2023)	62	Dual-tACS, dual-tDCS	20 Hz in-phase tACS, tDCS, sham (between subjects)	Right IFG	Six coordination blocks with stimulation in blocks 3 and 4	Higher INS in PFC for tACS compared to tDCS and sham in block 5; Higher INS in right IFG during rest period after stimulation for tACS compared to tDCS; Reduced activation in right IFG for tDCS during stimulation compared to poststimulation	Numbers of wins in blocks 3–6 higher than at baseline (block 1) in tACS and tDCS but not sham group, positive effect on number of wins in block 6 only for tACS not tDCS; Higher difference in reaction times (weaker coordination) in block 6 for tDCS compared to sham
Novembre et al. (2017)	30	Dual-tACS	frequency (within-subjects: 2 Hz, 10 Hz, 20 Hz) × relative phase (within-subjects: in-phase, anti-phase), sham stimulation	Left primary motor cortex	tACS during joint finger tapping task	NA	Increased synchrony for 20 Hz in-phase stimulation compared to anti-phase stimulation
Pan et al. (2021)	24	Dual-tACS	frequency (between-subjects: 6 Hz, 10 Hz) x relative phase (within-subjects: in-phase, antiphase, sham)	Left IFC	tACS during song learning task	NA	Increased synchrony for 6 Hz in-phase stimulation compared to 6 Hz sham stimulation; Improved intonation learning performance for 6 Hz in-phase compared to 6 Hz sham stimulation
Szymanski et al. (2017)	38	Dual-tACS	same-phase-same-frequency (6 Hz), different-phase-different-frequency (5 Hz and 7 Hz with 1 degree offset), 6 Hz sham (within-subjects)	Right frontal and parietal sites	tACS during joint drumming task	NA	Decreased synchrony for same-phase-same-frequency and the different-phase-different-frequency compared to sham

tACS, transcranial alternating current stimulation; tDCS, transcranial direct current stimulation; IFC, inferior frontal cortex; ATL, anterior temporal lobe; IFG, inferior frontal gyrus; PFC, prefrontal cortex; STG, superior temporal gyrus; N/A, not applicable.

While tACS may modulate the frequency of brain oscillations, e.g., in beta frequency band, tDCS does not target a specific frequency but induces subthreshold alterations in neuronal resting membrane potentials. In a tDCS protocol, stimulation was delivered to one participant of the dyad for 20 min. after which a resting-state session and social interaction task were conducted while measuring the brain activities using fNIRS hyperscanning (Long et al., 2023). Results showed that INS in the communication task was reduced after true compared to sham stimulation. Further, true stimulation decreased emotional empathy but importantly, the relationship between INS and emotional empathy was fully mediated through nonverbal behaviors. Thus, although there is ample correlational evidence for the importance of INS for learning, interpersonal understanding and affiliative bonding (e.g., Zheng et al., 2020; Zhang et al., 2022), the functional role of INS without behavior as a mediator is yet to be determined. Lu et al. (2023) compared the effects of 20 Hz in-phase tACS, tDCS and sham stimulation delivered to both participants over the right inferior frontal gyrus (rIFG) while their brain activities were measured simultaneous using fNIRS during a behavioral coordination task. They showed that tACS led to greater INS in the prefrontal cortex (PFC) for coordination after the stimulation while tDCS led to a reduced activation, indicating enhanced efficiency, in the rIFG. Importantly, tACS had longer lasting positive effects on behavioral coordination than tDCS. This shows that a comparison of tACS and tDCS can yield valuable insights into the unique effects of INS, yet these differences need to be interpreted with caution since effects may depend on parameter settings and intervention formulations, including the targeted cortex, intensity and frequency of stimulation (Lu et al., 2023).

To summarize, few studies exist examining the effects of brain stimulation on interpersonal synchrony. These studies have primarily focused on exploring the casual relationships between INS and behavioral synchrony and other outcome measures, however in some of the studies without directly assessing INS. Although some conflicting evidence exists, studies suggest that behavioral synchrony and related behavioral outcomes (e.g., coordination, learning, communication or conceptual understanding) may be enhanced by hyper-tACS.

#### 3.2.2 Hyperscanning based neurofeedback (hyper-NF)

Twelve publications including nine independent experimental data sets were identified that investigated the general feasibility and training success of hyper-NF using EEG (*N* = 10), fNIRS (*N* = 1), or fMRI (*N* = 1). Detailed information on methodological and design characteristics of the studies can be found in Boxes 1, 2. In a typical hyper-NF setup, the brain activations of two or more individuals are simultaneously measured to provide feedback on a shared target parameter (amplitudes or a measure of synchrony). This feedback allows individuals to collectively learn self-regulation of the target parameter. From a clinical perspective, the ultimate goal is to modify outcomes such as empathy, social affiliation or affective mental states. To assess whether participants were able to successfully regulate their brain activities / INS, we followed established regulation success classifications from single-person neurofeedback research (Thibault et al., 2018; Kohl et al., 2020). Specifically, we explored whether a study reported a significant effect as compared to a baseline control condition (CTB), for an early session as compared to a late session (ECTL), a linear increase (linear) and lastly if a study reported a significant effect as compared to a control condition (CTC). Results are depicted in Table 2 and study details provided in Supplementary Table S4. Findings are either based on the online analysis which was used for the calculation of the neurofeedback signal or an INS derived from an additional offline analysis.

**Table 2:** Hyper-NF study results.

Author	*N* dyads	Imaging modality	Target region	Task type	Regulation success as compared to			
					CTB	ECTL	Linear	CTC
Ceccato et al. (2023)	1	EEG	NR	Coop	Only 1 dyad and no statistics were reported			
Dikker et al. (2021)	784	EEG	Whole-brain	Coop	NR	Yes (offline^1^)	NR	Yes (offline^1^)
Duan et al. (2013)	1	fNIRS	Left sensorimotor cortex	Comp	Yes (offline^1^)	NR	NR	N/A
Järvelä et al. (2019)	21	EEG	Frontal cortex	Coop	NR	NR	NR	Yes
Järvelä et al. (2021)	39				NR	NR	NR	NR
Salminen et al. (2022) (incl. Overlapping data sets)	36				NR	NR	NR	NR
Kerr et al. (2022)	6	fMRI	Right anterior insular cortex	Coop	NR	NR	NR	N/A
Müller et al. (2021)	25	EEG	Frontal cortex	Coop	Yes	NR	NR	No
Putri et al. (2022) and Susnoschi Luca et al. (2021)^2^ (same data sets)	Comp.: 10 Coop.: 10	EEG	Parietal cortex	Both	**Comp**: Alpha power: no Synchrony (offline^1^): yes **Coop:** Alpha power: no Synchrony (offline^1^): yes	**Comp**: Alpha power: yes Synchrony (offline^1^): no **Coop:** NR	**Comp**: NR **Coop**: NR	**Comp**: NR **Coop**: NR
Vrins et al. (2023)	8	EEG	Whole-brain	Coop	Synchrony (offline^1^): yes Sham feedback (offline^1^): no	NR	NR	Yes (offline^1^)
Zhang and Zhao (2018)	1	EEG	Bilateral sensorimotor cortex	Comp	Only 1 dyad and no statistics were reported			

Comp, Competition; Coop, Cooperation; CTB, compared to a baseline control condition; ECTL, early session as compared to a late session; Linear, linear increase over several sessions; CTC, compared to a within- or between-control condition; NR, not reported; N/A, not applicable.

1 Some studies analyzed regulation success based on results from an additional offline analysis of neural synchrony which was different from the online analysis used to calculate the feedback signal.

2 Susnoschi Luca et al. (2021) did not compare synchrony to a within subject baseline, but used permutation testing against a null distribution created from fake pairs.

In a strictly controlled within-subject design, Müller et al. (2021) subjected participants to two different tasks in which they received real feedback, fake feedback (an enhanced signal meant to motivate the participants by giving them the impression that they are performing well) and inverted feedback (reinforcing desynchronization) of theta or delta frequencies (*N* = 25 dyads). Specifically, participants either had to move two balls as close as possible to each other by synchronizing their brain activities or they had to make two pendulums, each reflecting the oscillatory activity of one of the two participants, swing in phase. Across task and feedback conditions, participants demonstrated increased INS at theta and beta frequencies and partly also at delta frequencies compared to a resting state whereas INS at alpha frequencies decreased. Further validating their approach, they showed that INS at theta and beta was relatively strongly related to test partner likability and estimated ability to influence the feedback signal, as assessed using survey items. However, no significant differences between real and manipulated NF (enhanced and inverted) in INS emerged.

While in the study by Müller et al. (2021) both tasks were cooperative in nature, in the experiment by Susnoschi Luca et al. (2021) and Putri et al. (2022) participants controlled a virtual seesaw either in a cooperative or competitive interaction (*N* = 10 dyads each). Cooperating dyads had to maintain their Relative Alpha (RA) within 5% of each other to win shared points, while competing dyads won points if their RA was 10% above their opponent’s. The authors observed a decrease in alpha power in both competitive and cooperative tasks compared to rest. Further, significant INS in theta, alpha and beta frequency bands was found for both tasks, however, unfortunately cooperative, and competitive tasks were not directly compared in the study (Susnoschi Luca et al., 2021).

Three publications reported different analyses from the same experimental set-up (Järvelä et al., 2019, 2021; Salminen et al., 2022). In a virtual reality meditation environment, dyadic neurofeedback targeting EEG frontal asymmetry (neurophysiological measure of approach motivation) was combined with respirational biofeedback (*N* = 21, 39 and 36 dyads, respectively). Higher frontal asymmetry was observed in participants engaging in a dyadic neurofeedback condition compared to those in a solo neurofeedback condition. Additionally, increased synchrony of frontal asymmetry was observed when dyadic neurofeedback was combined with respiratory synchrony biofeedback, surpassing the effects of dyadic neurofeedback alone (Järvelä et al., 2019). Note, that the two later publications (Järvelä et al., 2021; Salminen et al., 2022) did not report respiratory or neural synchrony data. However, higher levels of empathy, emotion and social presence were reported following EEG-feedback and respiratory feedback, compared to a no-feedback condition, as well as after dyadic meditation compared to solo meditation conditions. Furthermore, the synchronization of EEG-frontal asymmetries between participants was associated with higher levels of empathy, with the highest ratings observed when both participants exhibited high values of frontal asymmetry (Järvelä et al., 2021; Salminen et al., 2022). Thus, these findings indicate that combining dyadic neurofeedback with biofeedback may augment both self-reported and neurophysiologically measured empathy.

Ceccato et al. (2023) built a dual brain-computer interface (BCI) that generates live music by measuring three EEG signal characteristics, which was adopted in a recent preprint by Vrins et al. (2023) to assess the effect of audio-based hyper-NF on interpersonal neural synchrony using a blinded protocol. Specifically, eight dyads (*N* = 4 dyads per group) listened to generated music. While in one group, three EEG signal characteristics (mean amplitude, frontal alpha asymmetry, and inter-brain phase-lock value) were mapped to the pitch, intensity, and consonance of the music, the control group was exposed to sham feedback based on randomly adjusted sound characteristics. The results showed a significant increase in INS through Hyper-NF compared to baseline only in the actual group, not in the control group. The authors also found that increased INS was associated with increased perceived synchrony and that the actual group reported a significant increased enjoyment, and changes in perceived mental state. However, it should be noted that participants anticipated ex-post correctly their group assignment. Hence, this study may indicate the potential of audio-based Hyper-NF.

In the so far largest hyper-NF study sample, Dikker et al. (2021) took a “crowdsourcing neuroscience” approach, in which they invited museum and festival visitors to sit in a “Mutual Wave Machine,” a dome-like neurofeedback environment that translates real-time correlations of each pair’s EEG activity into light patterns (final sample size: *N* = 784). Participants showed an increase in synchrony in the second compared to the first half of the experiment, however only if they were explicitly told that the visuals were derived from their correlated EEG signal. Further, this increase in synchrony in the second compared to first half was also observed in a sham feedback group, in which visualizations were randomly generated, indicating that the mere belief that the feedback signal was related to the success of the interaction could lead to higher social engagement irrespective of the actual relationship between inter-brain coupling and the feedback animation. Moreover, dyads´ relationship duration, social closeness, focus level, and social behavior (joint action and eye contact) positively and personal distress negatively predicted synchronization. While this crowdsourcing approach generated a very large sample, the authors also acknowledge its methodological limitations, in particular, the high likelihood of noise contamination, rendering it difficult to draw meaningful conclusions about the participants’ INS based on their online feedback.

Finally, three studies have developed and probed new experimental setups but with very limited samples sizes (*N* = 1–6; Duan et al., 2013; Zhang and Zhao, 2018; Kerr et al., 2022). Duan et al. (2013) developed a hyper-NF setup for fNIRS with a competitive “tug-of-war” game in which the target of both participants was to upregulate their left sensorimotor brain activities more strongly than the opponent in order to pull a ribbon to their side. Similarly, Zhang and Zhao (2018) developed a hyper-NF setup for EEG, testing it using a similar competitive “tug-of-war” game. Finally, Kerr et al. (2022) did not provide dyadic neurofeedback in the sense that the brain signals of two persons were fed back simultaneously but rather they probed whether mothers were able to downregulate the right anterior insular cortex signal of their adolescent daughter during an emotion discussion task.

To summarize, the field of hyper-NF is at a very early stage consisting mainly of proof-of-concept studies involving only limited sample sizes and oftentimes lacking stringent control conditions. These studies provide preliminary evidence that EEG-based hyper-NF is effective in modulating specifically targeted measures of INS. Not enough studies employing fMRI or fNIRS-based hyper-NF are available. While there is evidence that INS can be modulated by hyper-NF, neurofeedback based on amplitudes, based on a noisy signal or even sham neurofeedback may also enhance INS (Dikker et al., 2021; Müller et al., 2021).

### 3.3 *Clinical translation:* integration of INS-based manipulations in the treatment of mental disorders characterized by social interaction dysfunction

The three cases, presented in Box 3, although representing distinct clinical pictures, demonstrate the tremendous impact of social interaction dysfunction on our everyday functioning leading to severe impairments in building up trust and emotional connections in (close) relationships, in forming friendships with peers, and in overall academic development. Despite advancements in the last decade regarding our understanding of the neural basis of ASD, attachment issues, and anxiety disorders, the application of these insights into clinical practice remains largely confined. Over the past ten years, there have been few significant breakthroughs in the development of new therapeutic approaches, whether in psychotherapy or pharmacological treatments, for any of these mental conditions. Direct application of a transdiagnostic second-person neuroscience perspective-moving from a single brain towards at least two interacting brains—might open novel avenues for prevention and intervention of social interaction disorders.

BOX 3 **Clinical case presentations.**

Imagine the following scenarios:

A foster mother seeks psychotherapeutic help for her 2-year-old daughter Sally, whom she took into foster care 6 months ago due to neglect and suspicion of child abuse in her biological family. Sally seems withdrawn and anxious, shows no attachment to her foster parents, and does not play with other children in kindergarten. When she cries, Sally cannot be comforted by anyone, not even by the foster mother, who overall appears sensitive and empathetic when interacting with Sally.

Brian, a six-year-old boy, diagnosed with ASD three years ago, had been treated with intensive behavioral interventions, including applied behavioral analysis (ABA). Although his parents report that overall, Brian seems to profit from those interventions showing more adequate interactions within his family and with his little sister and less ASD-stereotypic behavior (i.e., less body rocking) he still suffers from marked social interaction deficits in the classroom. He typically misses the overall context of the lesson while focusing on specific details and he does not engage in any group activities which impairs both his integration into joint classroom activities with his peers as well as the acquisition of academic skills.

Susan, an 18-year-old university student, has always been shy and has consistently faced difficulties speaking in front of strangers. After moving to a new town for college, where she is unfamiliar with her peers and surroundings, she experiences a noticeable exacerbation of her social anxiety, including intense physical symptoms such as trembling, sweating, and palpitations as soon as she steps onto the campus. She seeks psychotherapeutic help as she is unable to attend her classes, participate in group projects, or engage in any typical social interactions on campus.

Coming back to the three cases presented above: Imagine, forming a secure attachment pattern between Sally’s foster parents and herself could be supported by engaging them in synchronous activities along with direct feedback to improve synchronization of their biological parameters in the caregiver-child dyad, such as synchronized oxytocin, cortisol levels, or simultaneous brain activities in those brain regions associated with mentalizing and trust. Imagine Brian’s learning difficulties in the classroom could be reduced by facilitating neural entrainment between the teacher and himself. For example, providing him with social ostensive signals that trigger transient moments of interpersonal teacher-pupil entrainment, in which the information presented by the teacher might arrive at a high receptivity phase for optimal encoding in Brian’s brain. And finally, imagine the effectiveness of a cognitive-behavioral therapy (CBT) for Susan’s social anxiety could be improved or shortened by synchronizing her ANS and neural activity with a socially non-anxious role model in prototype situations of social interactions.

In the following paragraphs, we will briefly describe what is already known about interpersonal synchrony, and INS in particular, in the three mental disorders, all characterized by social interaction dysfunction: ASD, RAD and SAD. We will summarize the current treatment standards according to clinical guidelines for each of these disorders and finally stimulate a critical discussion about the potential of tailored intervention to enhance INS in clinical treatment of these disorders and conclude with aspects of acceptability by participants and cost-effectiveness.

#### 3.3.1 Autism spectrum disorder (ASD)

ASD is a very heterogeneous, pervasive developmental disorder with life-long difficulties in social affect, including social interaction and communication, as well as restricted and repetitive behaviors. It affects approximately 1–3% of children and by the year of 2025 the cost of caring only for Americans with ASD will approximately reach up to 461 billion USD in the absence of more-effective interventions and support across the life span (Autism Statistics and Facts, 2023).

More recently, temporal synchrony has been in the focus of interest and might provide new insights for the understanding of social communication and sensory difficulties in ASD as experienced in everyday tasks and in naturalistic settings such as speaking back-and-forth on the telephone without visual cues, engaging in “flowing” one-to-one in-person conversation, and taking turns in social interactions. McNaughton and Redcay (2020) and Baldwin et al. (2022) reviewed existing studies on synchrony in ASD. Results demonstrated that participants with ASD tended to show more temporally asynchronous behavior when performing tasks that required audio-visual, audio-motor, visuo-tactile, visuo-motor, social motor, and conversational sensory integration.

In line with, a growing number of studies investigated interpersonal synchrony in ASD, and more recently also focused on INS (e.g., Lyons et al., 2020; Kruppa et al., 2021; Quiñones-Camacho et al., 2021; Wang et al., 2021; Key et al., 2022). Since these studies vary tremendously in the participants’ age (children, adolescents, and adults), as well as in tasks and imaging techniques (EEG, fMRI and fNIRS), it is difficult at this point to draw any strong conclusions. Kruppa et al. (2021) for instance found differences in behavioral synchrony between typically developing children and children with ASD, but no difference with respect to INS measures (i.e., wavelet coherence was calculated for oxy- and deoxyhemoglobin brain signals during a fNIRS task). However, using the same sample, Gerloff et al. (2022a) was able to predict ASD diagnosis based on non-linear connectivity estimators and network embeddings. Quiñones-Camacho et al. (2021) on the other hand, reported that healthy adults showed more neural synchrony than participants with ASD in the TPJ during a conversation task in an fNIRS study. Similarly, Key et al. (2022) reported that in typically developing adolescents and in adolescents with ASD, lower levels of synchrony, as measured with EEG, were associated with increased behavioral symptoms of social difficulties. Furthermore, Tanabe et al. (2012) used a real-time joint-attention task combined with dual-fMRI recordings and found that detecting gaze direction was impaired in both healthy subjects and subjects with ASD, when they were paired together and inter-brain coherence in the right inferior frontal gyrus (IFG) together with intra-brain functional connectivity between the right IFG and right superior temporal sulcus (STS) was diminished in ASD. These studies –although not completely consistent—point to impaired interpersonal synchrony across multiple levels in subjects with ASD.

Up to now, numerous interventions have been developed to improve ASD symptomatology. During early childhood, intensive behavioral interventions, including applied behavioral analysis (ABA, Cooper et al., 1987) and TEAACH (Mesibov et al., 2005), aim to improve difficulties in communication and social interaction. Nonetheless, its evidence is heterogenous, and randomized control trials (RCTs) are scarce. However, increasing evidence now supports the efficacy, and in some instances, the long-term impact of interventions focusing on parent–child interactions during the early years (e.g., European Child and Adolescent Psychiatry practice parameters). From school-age on, social skills training programs are commonly applied, mostly in high-functioning children with ASD, and just like early behavioral interventions, some evidence exists that show improvement in social functioning, including, i.e., social motivation, social anxiety, social cognition, and social skills (e.g., Spain and Blainey, 2015; Freitag et al., 2016). Nevertheless, so far, no interventions are available that can provide “cure” for ASD across the lifespan. In line with this, clinical guidelines refer to a multi-sensory, multi-disciplinary approach for the treatment of ASD (Subramanyam et al., 2019). Existing behavioral, psychosocial, educational, medical, and complementary approaches are recommended to be chosen based on the age and developmental status of the individual to maximize functional independence and quality of life by minimizing core deficits in social skills and communication, facilitating development and learning, promoting socialization, reducing maladaptive behaviors, and educating and supporting families. Intervention studies targeting interpersonal synchrony are still scarce yet, however, they provide first evidence for effectiveness and give hope for a whole new branch of intervention studies in ASD. Until now four interventions exist that target synchrony in individuals with ASD. Koehne et al. (2016) included 55 adults with ASD who either received 10 weeks of a dance movement intervention focusing on interpersonal movement imitation and synchronization (SI-DMI) or a control movement intervention (CMI). Patients in the SI-SMI group increased their synchronization skills and imitation tendencies, as well as whole-body imitation/synchronization and movement reciprocity/dialogue, compared to patients in the CMI group. In a RCT Landa et al. (2011) provided 50 toddlers with 10 h/week of classroom intervention, parent education setting (38 h) and 1.5 h of home-based parent training and instructional strategies. Additionally, half of the participants received a supplementary curriculum targeting socially engaged imitation, joint attention, and affect sharing. The authors report an increase in socially engaged imitation with eye contact in autistic toddlers, who participated in the supplementary curriculum. In a more recent study, Griffioen et al. (2020) provided six weekly sessions of 30-min-long dog-assisted therapy with children with ASD and children with Down’s syndrome focusing on psychomotor and socialization skills that would ensure aligned motor action between the child and the therapy dog. The authors also report an increase in synchronous interaction between children with ASD and their therapy dog. However, the number of participants (*N* = 10) in the study limits the generalizability of the findings. And last, in a pilot RCT (Srinivasan et al., 2015) 36 children with ASD between 5 and 12 years of age received one of three interventions (rhythm, robotic or standard-of-care) for eight weeks four times per week. In the rhythm and robot groups children got engaged in socially embedded whole-body movement games, whereas the children in the standard-of-care group engaged in tabletop activities promoting fine motor, social communication, and academic skills within a group setting. The rhythm and robot groups improved on the body coordination assessment, whereas the standard-of-care group improved on the fine manual control assessment. All three groups improved in imitation/praxis. The rhythm and robot groups also showed improved interpersonal synchrony performance.

These studies provide first and very preliminary evidence for behavioral joint-action interventions manipulating INS indirectly in dyadic interactions (person–person, child-dog, person-robot) in individuals with ASD. Other interventions to improve INS in ASD, such as neurofeedback or neurostimulation, have not been published yet. By contrast, neurofeedback interventions based on single-brain information using EEG, thus not including/targeting INS, have *not* been shown to be effective to improve ASD symptoms (Holtmann et al., 2011).

#### 3.3.2 Reactive attachment disorder (RAD)

RAD is characterized by absent or aberrant attachment behaviors in young children which are observable across settings. Children with RAD rarely interact with caregivers and they do not seek comfort or respond to comfort when distressed (American Psychiatric Association, 2013; Zeanah et al., 2016). In addition, they show limited or no positive affect, often appear socially and emotionally unresponsive and may display episodes of unexplained irritability, sadness, or fearfulness even if (familiar) adults are around. A child diagnosed with RAD should have a cognitive age of at least 9 months as this is the time, when infants usually have formed preferred attachments, and the symptomatology should develop before the age of 5 (American Psychiatric Association, 2013). RAD is a rather rare disorder, however in high-risk populations as, e.g., in children being raised in institutional care the prevalence rate is much higher and might reach up to 40% (O’Connor and Zeanah, 2003).

Children with RAD partly present similar symptoms as children diagnosed with ASD, e.g., social withdrawal and reduced social reciprocity. Both disorders are often associated with cognitive delays and stereotypes (Zeanah et al., 2016). The phenotypic similarity between the two disorders can be such that children exposed to sustained extreme early life institutional deprivation may present a symptom pattern that Rutter et al. (2007) termed “quasi-autism”. Importantly, in contrast to ASD, RAD is caused by the experience of adverse, neglectful caregiving environments including social neglect or deprivation, for example when being raised in institutions with high child-to-caregiver ratios or after having repeated changes of primary caregivers. As such, inadequate care is etiologic regarding the development of RAD as the deficits and the aberrant attachment behaviors develop in children who would have been able to develop functional social interaction processes if they had been raised in more favourable caregiving environments. Faced with severely limited opportunities to form selective attachments, affected children fail to develop attachments to any caregiver. However, it is important to bear in mind, that the RAD symptoms should not be reduced to a unique dysregulation of the children’s attachment system. Instead, the RAD symptoms rather seem to reflect a more general emotional and behavioral dysregulation (Rutter et al., 2009). This general pattern of dysregulation might be seen as a direct consequence of the lack of biobehavioral synchrony experience with a sensitive caregiver in early development. The important role of early synchrony experiences for the child’s social–emotional development has been shown in several studies. The quality of the early caregiver-child synchrony has been shown to predict the child’s later emotion and stress regulation capabilities, the expression of core social behaviors like engagement with peers and displaying empathy, as well as the exhibition of cognitive control (Feldman, 2012a, 2015b; Bell, 2020). Up to now, there are no studies investigating either behavioral synchrony or INS in children diagnosed with RAD. However, some studies considered biobehavioral synchrony in the context of insecure or disorganized attachment styles in high-risk caregiver-child conditions, such as in premature infants, in families who experienced multiple traumata or in mothers with postpartum depression (e.g., Feldman, 2015a; Granat et al., 2017; Pratt et al., 2019; Ulmer-Yaniv et al., 2023). For instance, it could be shown that depressed mothers and their infants were less synchronous, i.e., had lower levels of gaze and touch synchrony, reduced coordination of affectionate touch with mutual gazing, and diminished maternal behavior (Granat et al., 2017). Pratt et al. (2019) investigated the impact of early and persistent maternal depression on the child’s brain and general development in a prospective longitudinal study. They not only found an increased prevalence of affective disorders in these children, but also a longitudinal impact of maternal depression on the children’s oxytocin levels across early childhood and aberrant neural responses to attachment-specific and social cues in preadolescence.

Research regarding the relationship between attachment quality and INS is still in its infancy (Long et al., 2020) and so far, studies with children with RAD or with high-risk dyads are missing. Using fNIRS hyperscanning in 28 mother–child dyads during the performance of a cooperation task Miller et al. (2019) found some preliminary evidence in an ROI-based correlation analysis that avoidant attachment in the child was associated with reduced INS in the right frontopolar PFC. Another fNIRS hyperscanning study in 42 dyads of mothers and their preschool children demonstrated that INS was positively correlated with behavioral reciprocity (Nguyen et al., 2020).

With regard to treatment, the clinical guidelines for RAD recommend as treatment of first choice the child’s placement in a safe and sensitive caregiving environment with an emotionally available attachment figure as early as possible to minimize the consequences of the psychosocial deprivation on the child’s development (Practice Parameter of the American Academy of Child and Adolescent Psychiatry (AACAP); Zeanah et al., 2016). However, due to a lack of research in this field, it is less clear whether children who recover from RAD still have an enhanced risk for subsequent interpersonal difficulties. To help the child and the caregivers to attune to each other and to interact more positively, the AACAP-Parameter additionally recommend psychotherapeutic interventions targeting the caregiver-child-dyad (Zeanah et al., 2016). Among them the child–parent psychotherapy (CPP; Lieberman, 2004) and the Attachment and Biobehavioral Catch Up intervention (ABC; Bernard et al., 2012; Dozier and Bernard, 2017) were found to be effective in RCTs, though in samples of children with disturbed attachment relationships, not specifically in toddlers with attachment disorders. ABC, a manualized, in-home treatment program includes synchrony-based interventions and is targeted to “catch up” the missed experience of nurturing and sensitive caregiving. Consequently, ABC focuses on directly changing caregivers’ behaviors by, e.g., using direct behavior-focused in-the-moment comments when caregivers interact with the child as well as video feedback. Three main parenting targets are addressed during the intervention, namely nurturing the child when distressed, following the child’s lead (synchronous behavior), and behaving in nonfrightening ways (Dozier and Bernard, 2017). In ABC-T for toddlers the third target is replaced by helping the caregivers to serve as co-regulators when toddlers show signs of dysregulation as, e.g., anger or frustration or aggressive behavior (Lind et al., 2017; Imrisek et al., 2018). It could be shown that the ABC intervention led to enhanced caregivers’ synchronous behaviors and improved attachment behavior, cortisol production, and executive functioning in the child (Dozier et al., 2013).

These first studies addressing the enhancement of biobehavioral synchrony as a core intervention target provide evidence that attachment- and synchrony-based interventions can help to attenuate the consequences of psychosocial deprivation. However, treatment interventions directly targeting INS in socially deprived children have not been published yet.

#### 3.3.3 Social anxiety disorder (SAD)

SAD is a persistent anxiety and avoidance reaction triggered by social or performance situations. SAD may escalate to panic attacks when facing fearful situations such as speaking or eating in the presence of others or engaging in social activities. Individuals with SAD experience distress and/or impairment in occupational and social functioning. SAD often becomes chronic without intervention. The anxiety reaction typically extends to all social contexts and varies in severity, influenced by stressors and life changes (Stein and Stein, 2008; Steinert et al., 2013). SAD is highly prevalent, with a lifetime and 12-month prevalence of 13 and 8%, respectively, among adults in the United States and similar rates in adolescents (Kessler et al., 2005, 2012; Kessler and Üstün, 2008). Onset is typically during adolescence (mean age: 13 years) and rarely after age 25 (Stein and Stein, 2008; Steinert et al., 2013).

Given the considerable difficulties individuals with SAD face in interpersonal scenarios, investigating interpersonal synchrony within this patient group holds substantial promise. Asher et al. (2020) recently examined behavioral and heart rate (HR) synchrony during closeness-generating and small talk conversation in opposite-sex dyads including subjects with and without SAD. It was shown that closeness-generating conversation compared to small-talk conversation led to increased behavioral synchrony (as derived from computer-based video analysis) in control dyads but not in subjects with SAD. Furthermore, the authors found that during intimate conversations, social anxiety correlated with increased HR synchrony in control dyads but decreased HR synchrony in SAD dyads, suggesting that SAD may hinder the ability to establish HR synchrony in closer social contexts, potentially affecting relationships negatively (Asher et al., 2021). So far, INS has been hardly addressed in the context of SAD. However, one study conducted an EEG hyperscanning study with parent-adolescent dyads, revealing that SAD in adolescents is associated with heightened gamma interbrain synchrony in positive emotional contexts and diminished gamma interbrain synchrony in emotionally negative context during socioemotional interactions (Deng et al., 2022).

Clinical guidelines for the treatment of SAD (e.g., Germany: S3 guideline, Bandelow et al., 2014); United Kingdom: NICE guidelines (National Institute for Health and Care Excellence, 2013); Canada: Canadian Clinical Practice Guidelines (Katzman et al., 2014) recommend both, cognitive behavioral therapy and pharmacotherapy for treatment. Several RCT studies in subjects with SAD demonstrated high efficacy for exposure therapy and cognitive restructuring (effect size: 1.8) and for selective serotonin reuptake inhibitors (effect size: 1.5) (Fedoroff and Taylor, 2001; Stein et al., 2004; Canton et al., 2012). While divergent study designs hinder direct effect size comparisons between psychotherapy and pharmacotherapy, emerging evidence suggests faster effects with pharmaceuticals and potentially more enduring effects with CBT (Gelernter et al., 1991; Heimberg et al., 1998; Liebowitz et al., 1999; Haug et al., 2003; Davidson et al., 2004; Nordahl et al., 2016). RCTs comparing monotherapies with combined CBT and psychotropics yield heterogeneous results (Knijnik et al., 2008; Stein and Stein, 2008; Canton et al., 2012; Baldwin et al., 2014; Nordahl et al., 2016).

Moreover, given that individuals with SAD inherently fear social interactions, the question emerges about the potential of therapeutic approaches aimed at reinforcing synchronization for therapeutic efficacy. As of now and to our knowledge, no study has explicitly targeted synchronization between therapist and patient with SAD, despite its assessment as an outcome measure in previous research. Altmann et al. (2020) investigated the effect of movement synchrony in interactions between individuals with SAD and psychotherapists, without putting synchrony in the therapeutic focus. The study revealed that heightened synchrony led by the therapist correlated with improved therapeutic alliance and decreased interpersonal issues. Similarly, Schoenherr et al. (2019) found that therapist-patient pairs, which included patients with SAD who prematurely terminated psychotherapy, exhibited lower movement synchrony at the therapy onset compared to patients who completed therapy. Ramseyer and Tschacher (2011) reported analogous results across more diverse disease profiles. Notably, if patients took a leading role in synchrony at the end of the psychotherapy, a reduced therapeutic alliance was observed along with increased scores for interpersonal problems and depression (Altmann et al., 2020). Furthermore, studies on vocal synchrony in patient-therapist interactions (Schoenherr et al., 2021) revealed that heightened patient-led vocal synchrony correlated with elevated symptom severity, attachment anxiety, avoidance, and interpersonal problems in subjects with SAD.

Previous studies have demonstrated that the inclusion of neurofeedback as a treatment tool can produce effects in reducing symptoms in generalized anxiety disorder (Hou et al., 2021) and specific phobia (Zilverstand et al., 2015). In an initial pilot study, NIRS-based neurofeedback targeting the dlPFC in individuals with SAD was deemed feasible (Kimmig et al., 2019). Participants improved dlPFC control, altering social threat-related attention bias. Decreased social anxiety severity was linked to reduced attentional threat processing and successful NF training. NIRS-based NF offers potential to explore attention biases and the dlPFC’s role in SAD. Nevertheless, subsequent research should encompass larger sample sizes and suitable control groups to validate and extend these initial findings. While these studies focused on a single-subject, Saul et al. (2022) were the first to introduce hyper-NF protocol (called “InBS-NF-paradigm”) for clinical application in SAD employing multi-user neurofeedback for intervention. The paradigm aims to integrate neural activities of interacting partners with external sources to minimize subjective distortions. It enables real-time observation and treatment of SAD symptoms by updating the mental self-representation using objective external cues. This work exemplifies the first attempts to directly target INS in clinical treatment.

### 3.4 Feasibility of neuroscience-based intervention approaches in patients with social interaction disorders

If interventions that directly target the neural basis of a mental disorder in at least two interacting individuals, e.g., by hyper-NF or by brain stimulation, could represent novel treatment options, implementation depends not only on the effectiveness of such approaches but is also heavily influenced by the patients’ perspective on tolerability and acceptance of such treatment approaches as well as cost-effectiveness and safety of the intervention. The systematic database search for feasibility of neurofeedback and neurostimulation approaches in subjects with social interaction disorders provided overall good support for such methods (see Table 3 for study results). NF studies in patients with social interaction disorders reported no clinically meaningful adverse events. Generally, both EEG and fNIRS, typically used in NF, are considered safe and can be used in all patient groups (Strehl et al., 2017; Pinti et al., 2020). Similarly, non-invasive brain stimulation methods used in the therapy of mental disorders, such as tDCS, trigeminal nerve stimulation (TNS) or transcutaneous electrical nerve stimulation (TENS), are well tolerated with mild side effects like skin irritations from electrodes (Matsumoto and Ugawa, 2017; Wei et al., 2017). One neuromodulation study conducted with participants with social interaction disorders used TENS and reported no adverse events (Foldes et al., 2021). Three studies used tDCS in patients with ASD and reported no adverse events as well as low drop-out rates (D’Urso et al., 2015, 2022; Wang et al., 2023).

**Table 3:** Study results for feasibility of neuroscience-based intervention approaches for social interaction disorders.

Author	Patient group	*N*	Intervention	Outcome measures	Results
Direito et al. (2021)	ASD (16-22y)	15	NF (fMRI, facial expression task)	Fidelity; feasibility; acceptability	All measures 100%; 1 drop-out
D’Urso et al. (2015)	ASD (18-26y)	12	tDCS	Drop-out rate	No adverse events; 2 drop-outs
D’Urso et al. (2022)	ASD (9-13y)	7	tDCS	Drop-out rate	No adverse events; no drop-outs
Foldes et al. (2021)	ASD (10-21y)	7	Transdermal electrical neuromodulation	Adverse events (AE); tolerability (sham-controlled)	No significant AEs reported during the trial; no participant reported discomfort from the study procedures (including the electrodes)
Kimmig et al. (2019)	SAD (over 18y)	12	NF (NIRS, social threat-processing experiment)	Enjoyment; importance of performing; effort they put into the training; if they would recommend it	2 drop-outs; 9 would recommend it to others, 3 were unsure; motivation to perform well: *M* = 3.58, *SD* = 0.52; enjoyment of training: *M* = 3.00, *SD* = 0.74 (scale was 0 to 4)
LaMarca et al. (2018)	ASD (6-8y)	8	NF (EEG, TAGteach-assisted NF)	Case study; description of fidelity, feasibility, and acceptability for each case	Method is feasible; some patients showed difficulties with focus fatigue, disruptive behavior, and tolerability of sensory aspects of NF, but issues could be resolved by the instructor
Steiner et al. (2014)	ASD (7-12y)	10	NF (EEG, play attention & academic tasks)	Motivation; feedback of intervention questionnaire (parents & child)	All children reported it was easy to understand, follow, and that the sessions helped them concentrate; 3 had low motivation scores; 5 said sessions were “boring”; 90% of parents reported that the intervention was helpful
Wang et al. (2023)	ASD (4–12 y)	45	tDCS	Adverse events	With adaptations, all children tolerated the treatment; all children developed scalp erythema, but it disappeared after 30 min; 3 had symptoms of excitement and night terrors which improved on their own; no serious AEs occurred

ASD, Autism Spectrum Disorder; SAD, Social Anxiety Disorder; NF, Neurofeedback; tDCS, transcranial direct current stimulation.

In NF studies, acceptability was high. In a study by Kimmig et al. (2019), 9 out of 12 of participants in a NF training for social anxiety disorder would recommend the treatment. A fMRI-based NF study examining adolescents with ASD reported high fidelity, feasibility, and acceptability (Direito et al., 2021). Some studies reported that children had difficulty concentrating on the NF task (Steiner et al., 2014; LaMarca et al., 2018). LaMarca et al. (2018) conducted an EEG-NF intervention with children with ASD and reported that some participants displayed problems with concentration, compliance as well as sensory issues associated with the NF protocol (e.g., electrode placement, noises).

Note, however, that the studies included here had few participants and reported on few measures of acceptability. However, the findings are in line with NF studies in other patient groups. A meta-analysis from Catalá-López et al. (2017) found lower drop-out rates for NF treatment for children and adolescents with ADHD compared to cognitive training, and no difference to a wide range of other pharmaceutical and non-pharmaceutical treatments, suggesting high acceptability.

While acceptability of single-brain-based neuroscience-based interventions was high in patients with social interaction disorders, this cannot be automatically generalized to dual-brain-based interventions. Targeting the INS requires interventions of social interacting partners. This could pose an additional challenge. Therefore, future studies need to specifically assess acceptability for neuroscience-based interventions involving synchrony.

### 3.5 Considerations of cost-effectiveness of neuroscience-based interventions in patients with social interaction disorders

In addition to acceptability and safety, cost-effectiveness is a crucial factor for the implementation of any novel treatment option. Commonly used behavioral therapies for patients with ASD are costly, e.g., ABA-based interventions are estimated to cost around 45,000 USD annually per patient (Hodgson et al., 2022). Treatments for social anxiety disorders, such as cognitive behavioral group therapy, typically amount to approximately 2,500 USD for a 15-session program consisting of two-hour sessions (Stuhldreher et al., 2014). Cost for neuroscience-based interventions using hyperscanning depend on the specific protocol and the type of neuroimaging method that is used. In an EEG NF study, Arnold et al. (2013) reported that after 24 sessions, the parent rating of ADHD symptom improvement plateaued. Assuming a similar number of treatment sessions for NF treatment of social interaction disorders and calculating the costs for one-person NF intervention (according to the remuneration of the German health insurance funds for NF training), the total cost of one-person NF intervention is around 1,500 USD (for 24 sessions). Two-person NF requires two neuroimaging devices, but no additional therapeutic personnel are needed, so the cost of each session for hyper-NF should be comparable to one-person NF. In contrast, fMRI-based hyper-NF constitutes an exception as it requires twice costly scanner time and additional technical personnel. Recent developments of low-cost neuroimaging devices for EEG (Niso et al., 2023) and fNIRS (Vidal-Rosas et al., 2023) have increased accessibility to mobile NF equipment. Taken together, compared to standard treatment of social interaction disorders, NF training based on EEG and fNIRS can be considered relatively low-cost. Thus, such approaches might yield high potential to develop cost-effective interventions, however, as a first step, the field requires a robust evaluation of the effectiveness of such non-pharmacological interventions.

## 4 Conclusion

Despite their preliminary nature, early findings outline the potential of INS-based manipulations, such as hyper-NF. Although predominantly conducted in healthy adult cohorts in the past, the findings motivate the extension of this technique to clinical and developmental samples, along with further developments of methodological approaches. Hyper-NF may exhibit greater transferability to everyday situations, particularly through its association with social experience and learning, compared to traditional neurofeedback approaches which have often struggled to demonstrate substantial real-world transfer (Kohl et al., 2020). Even other innovative and motivating treatment options, such as “serious games”, may not fully address this aspect (Dewhirst et al., 2022). In contrast, targeting directly the INS might be distinct, as it couples learning mechanisms with physiological processes, linking a natural synchronization process with a conditioned stimulus.

Nevertheless, for clinical applications, first several technical and data analytical challenges must be solved. In particular, well-controlled studies with larger samples are needed to determine the most suitable hyper-NF parameters, such as experimental task and instructions, target brain regions and feedback signal, presentation of the feedback signal, required number of trials, runs and sessions etc. (see Boxes 1, 2). In addition to neurofeedback control conditions, e.g., sham neurofeedback, it will be key to compare feedback of neural synchrony to synchrony feedback in other modalities, such as heart rate or breathing (see also Järvelä et al., 2019, 2021; Salminen et al., 2022), as well as to behavioral interventions to determine the most effective technique or combination of techniques for enhancing INS. Furthermore, task and hyper-NF settings have to be evaluated with respect to their transferability. For instance, for patients with SAD, a training that involves no or little direct interaction between participants (e.g., back-to-back setting, Müller et al., 2021) may be less suitable although it allows for better experimental control. Similarly, for dual-brain stimulation more methodological groundwork is needed. In particular, previously conflicting findings suggest that identifying the optimal stimulation protocol, e.g., with respect to the specific frequencies and stimulation sites, will be crucial for developing this technique further. In addition, it will be necessary to assess short- and long-term changes in INS in response to single and repeated stimulation. In this review, we outline the importance of tailored design and method considerations (see Section 3.2.2), as well as key factors to report (see Supplementary Table S4) when combining hyperscanning with interventional techniques. Moving forward, future work may substantially benefit from an improved mechanistic understanding of INS (Lotter et al., 2023), e.g., to efficiently target relevant brain regions, as well as from further developments of technique-specific designs, reporting standards (Ros et al., 2020) and methods and from fostering the application of open science practices (Allen and Mehler, 2019; Niso et al., 2022; Botvinik-Nezer and Wager, 2023).

Based on the evidence of the current review, one can conclude that although direct strategies have shown some promise for improving INS, there is no concrete evidence that the effects of direct stimulation are more substantial than those achieved through indirect strategies. Furthermore, so far, no data are available how long such direct effects might last. Thus, given that few studies have attempted a comparison between direct and indirect INS-based manipulations (for exceptions see Järvelä et al., 2019, 2021 and Salminen et al., 2022) and none have examined their transferability, no clear conclusions can be drawn about the efficiency of direct versus indirect techniques. Interestingly, the findings of Järvelä et al. (2019) indicate that a combination of different types of feedback (here EEG neurofeedback and respiratory-based biofeedback) could potentially surpass the effects of one type of feedback alone. This is in line with many other areas of clinical applications of direct brain manipulations, such as in depression (Xu et al., 2023) neurological rehabilitation (Xu et al., 2022) or pain management (Goudra et al., 2017). In this regard it should also be noted that not all patients may profit equally from different types of interventions. Thus, identifying which types of interventions work best for certain groups of individuals (see also “precision medicine”) may be an important avenue for future research.

The current review emphasizes the unique role of INS in selected mental disorders characterized by core deficits in social interactions. However, as outlined in this review, before INS-based interventions can be applied in clinical contexts there is quite a long way to go from the current state of knowledge towards RCTs testing the efficacy of INS-based interventions either as a stand-alone or as an add-on intervention to the current standard treatment approaches of the respective disorder. For example, in the case of ASD, future trials need to demonstrate that combined approaches including social competence trainings together with targeted interventions incorporating Hyper-NF or dual neurostimulation might be more effective than social competence training alone to facilitate not only cognitive but also affective empathy, thus creating a more holistic approach to managing social interaction dysfunctions in subjects with ASD.

Clinical applications based on a second-person neuroscience approach must be built on specific dysfunction in interpersonal synchrony. They should target those modalities of synchrony that have the largest effects to achieve the “optimal” or the “most healthy state” (see also Gordon et al., 2023). Gordon et al. (2023) proposed an alternative theory of flexible multimodal synchrony which highlights the context as a key component that defines “pulls” towards synchrony and “pulls” towards segregation inherent to the social situation. In the case of the mental disorders described above, this aspect becomes particularly relevant in the context of allostasis co-regulation in relationships, such as in the mother–child bond in RAD or in the patient-therapist-relationship in SAD. For instance, if a mother is comforting her crying child, it is intuitive to assume that maximal synchrony will not be most goal-conducive but that a temporary “desynchronization” may be more beneficial instead which allows the mother to down-regulate the child’s affective state (Long et al., 2020). Thus, clinical intervention that include a monitoring of functional and dysfunctional synchrony patterns in interacting partners should enable and support the participants not only to pull towards synchrony but also to push out of maladaptive interpersonal synchrony. Furthermore, as there is growing evidence that synchrony of body and mind is distinct and their relationship is dependent on context (Reindl et al., 2022), a second challenge concerns the identification of the most efficient target of the different (i.e., behavioral, physiological, endocrine, and neural) measures of interpersonal synchrony. For example, increasing INS between caregivers and their children might support mentalizing and empathy towards their children’s needs while ANS synchrony in some situations might be associated with higher overall distress in the dyads. Finally, for clinical translations, selecting the best patient’s partner for INS-based intervention, e.g., a therapist, romantic partner, peer or the child’s primary caregiver, will be crucial for treatment success and strongly depend on disorder-specific social dysfunction.

While further research is necessary to fine-tune these interventions and establish their efficacy, this exploration of interpersonal synchrony paves the way for promising advancements in the realm of mental health which may—in principle—be relevant for many other mental disorders as well as for neurological diseases that are characterized by impaired social interactions.

## Data availability statement

The original contributions presented in the study are included in the article/Supplementary material, further inquiries can be directed to the corresponding author.

## Author contributions

KK: Conceptualization, Funding acquisition, Resources, Supervision, Writing – original draft, Writing – review & editing. CG: Methodology, Software, Visualization, Writing – original draft, Writing – review & editing. SK: Methodology, Writing – original draft, Writing – review & editing. DM: Writing – original draft, Writing – review & editing. LM: Visualization, Writing – original draft, Writing – review & editing. EV: Methodology, Writing – original draft, Writing – review & editing. MK: Writing – review & editing. AH: Writing – original draft, Writing – review & editing. MB: Writing – original draft, Writing – review & editing. EW: Writing – original draft, Writing – review & editing. VR: Conceptualization, Methodology, Resources, Supervision, Writing – original draft, Writing – review & editing.
