# Supplementary material for: Interpersonal neural synchrony and mental disorders: unlocking potential pathways for clinical interventions
Source: Front Neurosci. 2024 Mar 11;18:1286130. doi: 10.3389/fnins.2024.1286130 (PMC10962391; doi:10.3389/fnins.2024.1286130)
Supplement: Supplementary file 2 [file Data_Sheet_1.PDF]

Supplementary Material

1 Structured Literature Search

The structured literature search was conducted using the open-source search engine SetYouFree (v0.1.2 Gerloff et al. 2022), which follows best practices of the PRISMA guidelines for systematic reviews. Detailed search results can be found in SI Files S1-S3 (JSON file; https://gin.g-node.org/ChristianGerloff/INS-disorders-publication) and Table S4 (Excel table).

As most direct targeting techniques are in their early stages, the search was performed across scientific publication servers as well as preprint servers using harmonized search strings. Duplicate publications and commentaries were automatically merged based on the similarity of abstracts and titles calculated from the enhanced version of the Ratcliff-Obershelp algorithm. Furthermore, publications were merged with their respective preprints. Automatic keyword or citation based exclusion was not performed. The main search parameters for each systematic search can be found in the following tables.

1.1 Brain Stimulation

Supplementary Table S1. Search Parameters: Brain Stimulation

Parameter	Value
Databases searched	ACM, arXiv, bioRxiv, IEEE, medRxiv, Pubmed, Scopus
Search string	[neurostimulation] OR [TMS] OR [tDCS] OR [tACS]) AND ([synchrony] OR [interpersonal synchrony] OR [interpersonal neural synchrony] OR [interbrain] OR [hyperscanning] OR [brain-to-brain] OR [cross-brain] OR [dyad] OR [two-person] OR [triad] OR [three-person]
Publication types	Book chapters, publications, conference proceedings, preprints including: methodological studies, empirical studies, introduction of concepts, pilot and proof-of-concept studies, randomized controlled trials
Start date	2000-01-01
End date	2023-12-31
Conducted at	2024-01-05
Keyword filter	None
Citation filter	None
Similarity threshold	0.9
Exclusion criteria	No brain stimulation involved, less than two participants, no manipulation of interbrain synchrony, reviews, non-human studies, not available in English

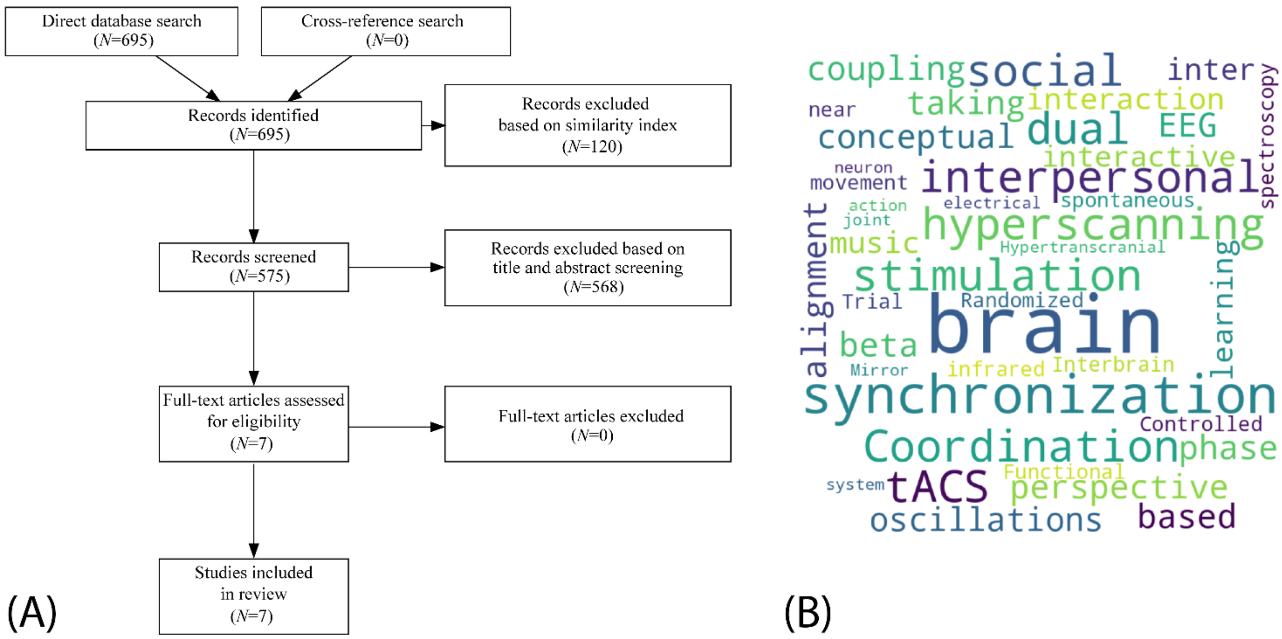

Supplementary Figure S1. Search Details: Brain Stimulation. (A) shows the flow diagram rendering the step-by-step progression of the structured literature following PRISMA guidelines, from initial identification of studies through to final inclusion in the review. (B) shows the word cloud of the structured search including prominent keywords. The size of each keyword corresponds to its frequency of occurrence within the dataset. Common themes emerge as larger, bolder words.

1.2 Neurofeedback based on hyperscanning

Supplementary Table S2. Search Parameters: Neurofeedback based on hyperscanning

Parameter	Value
Databases searched	ACM, arXiv, bioRxiv, IEEE, medRxiv, Pubmed, Scopus
Search string	([neurofeedback] AND [synchrony]) OR ([neurofeedback] AND [interbrain]) OR ([neurofeedback] AND [hyperscanning]) OR ([neurofeedback] AND [interpersonal synchrony]) OR ([neurofeedback] AND [interpersonal neural synchrony]) OR ([neurofeedback] AND [brain-to-brain]) OR ([neurofeedback] AND [cross-brain]) OR ([neurofeedback] AND [dyad]) OR ([neurofeedback] AND [two-person]) OR ([neurofeedback] AND [triad]) OR ([neurofeedback] AND [three-person])
Publication types	Book chapters, publications, conference proceedings, preprints including: methodological studies, empirical studies, introduction of concepts, pilot and proof-of-concept studies, randomized controlled trials
Start date	2000-01-01
End date	2023-12-31
Conducted at	2024-01-08
Keyword filter	None
Citation filter	None
Exclusion criteria	No neurofeedback involved, no hyperscanning setup, less than two participants, no measure of interbrain synchrony as a target parameter or outcome parameter, reviews, non-human studies, not available in English

Inter-rater reliability (κ)	0.95

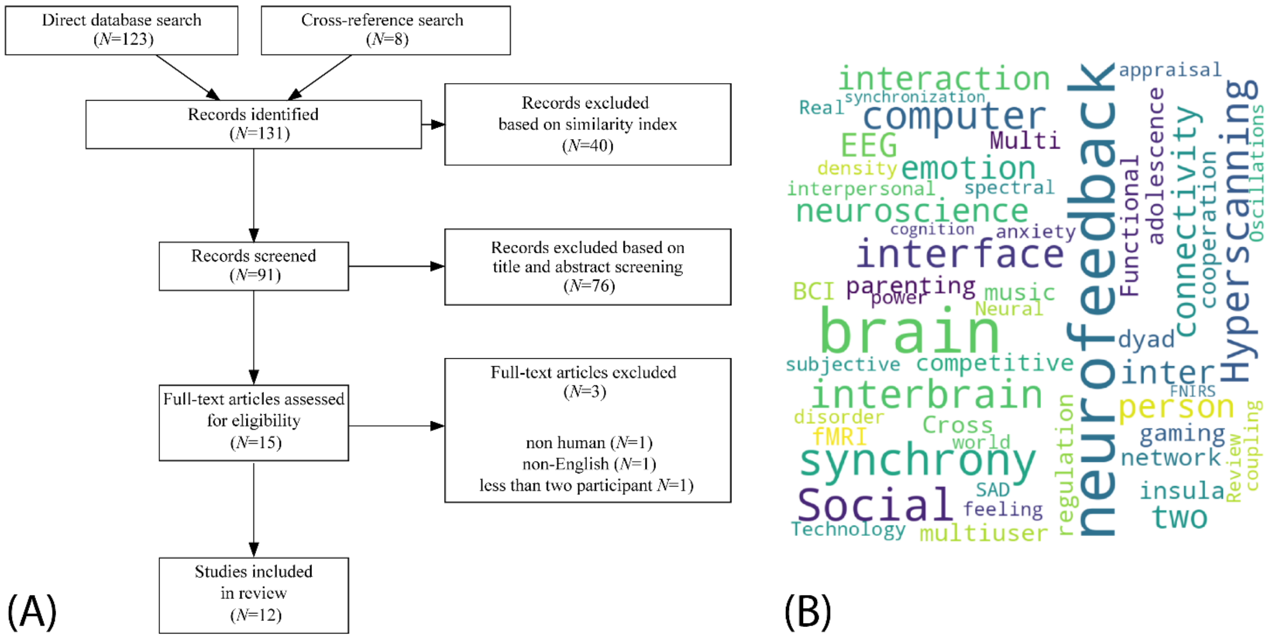

Supplementary Figure S2. Search Details: Neurofeedback based on hyperscanning. (A) shows the flow diagram rendering the step-by-step progression of the structured literature following PRISMA guidelines, from initial identification of studies through to final inclusion in the review. (B) shows the word cloud of the structured search including prominent keywords. The size of each keyword corresponds to its frequency of occurrence within the dataset. Common themes emerge as larger, bolder words.

1.3 Acceptability and Feasibility

Supplementary Table S3. Search Parameters: Acceptability and Feasibility

**Parameter**	**Value**
Databases searched	ACM, arXiv, bioRxiv, IEEE, medRxiv, Pubmed, Scopus
Search string	([neurofeedback] OR [neurostimulation] OR [TMS] OR [tDCS] OR [tACS]) AND ([social] OR [autism] OR [attachment]) AND ([acceptability] OR [feasibility] OR [acceptance] OR [compliance] OR [adherence])
Publication types	Book chapters, publications, conference proceedings, preprints including: methodological studies, empirical studies, introduction of concepts, pilot and proof-of-concept studies, randomized controlled trials
Start date	2000-01-01
End date	2023-12-31
Conducted at	2024-01-10
Keyword filter	None
Citation filter	None
Exclusion criteria	no patients with social disorders (SAD, ASD or attachment disorders), no concise reports on feasibility, non-human studies, not available in English, review

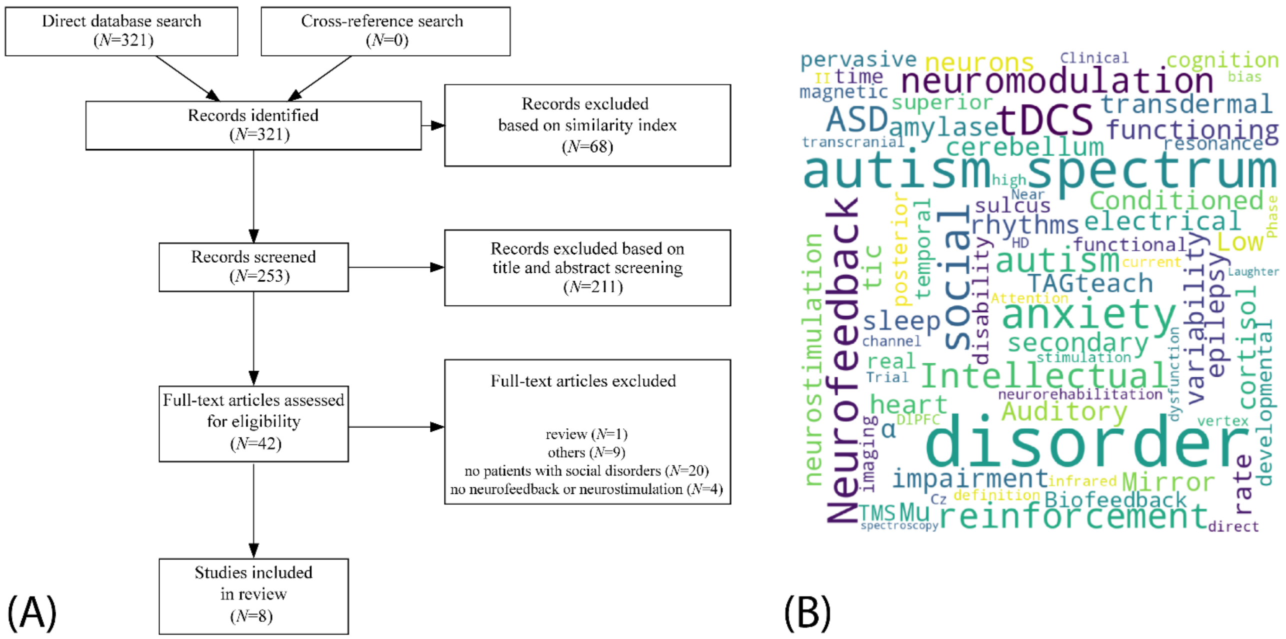

Supplementary Figure S3. Search Details: Acceptability and Feasibility. (A) shows the flow diagram rendering the step-by-step progression of the structured literature following PRISMA guidelines, from initial identification of studies through to final inclusion in the review. (B) shows the word cloud of the structured search including prominent keywords. The size of each keyword corresponds to its frequency of occurrence within the dataset. Common themes emerge as larger, bolder words.
